# ATAC‐seq in Emerging Model Organisms: Challenges and Strategies

**DOI:** 10.1002/jez.b.23305

**Published:** 2025-06-01

**Authors:** Duğçar Ebrar Erdoğan, Shadi Karimifard, Mozhgan Khodadadi, Liucong Ling, Luisa Linke, Ana Catalán, Vincent Doublet, Amanda Glaser‐Schmitt, Oliver Niehuis, Katja Nowick, Antonella Soro, Natascha Turetzek, Barbara Feldmeyer, Nico Posnien

**Affiliations:** ^1^ Department of Developmental Biology, Göttingen Center for Molecular Biosciences (GZMB) University of Göttingen Göttingen Germany; ^2^ Evolutionary Ecology, Faculty of Biology, Biocenter Ludwig‐Maximilians‐University of Munich Planegg‐Martinsried Germany; ^3^ Molecular Ecology, Senckenberg Biodiversity and Climate Research Centre Frankfurt am Main Germany; ^4^ Institute of Organismic and Molecular Evolution Johannes Gutenberg University of Mainz Germany; ^5^ General Zoology, Institute for Biology Martin Luther University Halle‐Wittenberg Halle Germany; ^6^ Institute of Evolutionary Ecology and Conservation Genomics University of Ulm Ulm Germany; ^7^ DGIMI Univ Montpellier, INRAE Montpellier France; ^8^ Evolutionary Biology, Faculty of Biology, Biocenter Ludwig‐Maximilians‐University of Munich Planegg‐Martinsried Germany; ^9^ Department Evolutionary Biology and Ecology, Institute of Biology I (Zoology) University of Freiburg Freiburg Germany; ^10^ Institute for Biology Freie Universität Berlin Berlin Germany

**Keywords:** ATAC‐seq, benchmarking, chromatin accessibility, gene regulation, insects, protocol optimization, quality control, spiders, tissue preservation

## Abstract

The Assay for Transposase‐Accessible Chromatin with sequencing (ATAC‐seq) is a versatile and widely utilized method for identifying potential regulatory regions, such as promoters and enhancers, within a genome. ATAC‐seq has been successfully applied to a wide range of established and emerging model organisms. However, implementing this method in emerging model systems, such as arthropods, can be challenging due to several factors that influence data quality. These factors include the availability of a sufficient amount and quality of tissue or cells, the need for species‐ and tissue‐specific protocol optimization, the completeness and accuracy of the reference genome, and the quality of the genome annotation. In this article, we emphasize the key steps in the ATAC‐seq protocol that, based on our experience, have the greatest impact on data quality when adapting this method for emerging model organisms. Specifically, we discuss the importance of nuclei isolation, the incubation conditions of the Tn5 transposase, and PCR amplification of the library. Furthermore, we outline essential quality checkpoints during the bioinformatic analysis of ATAC‐seq data to assist in assessing data integrity and consistency. Given that many emerging model organisms may not be readily available in laboratory cultures, we also emphasize the importance of evaluating how different preservation methods affect ATAC‐seq data quality. Based on examples in one spider and one ant species, we demonstrate that replication and thorough quality controls at all steps of the protocol and data analysis are essential to assess the usability of ATAC‐seq data. Our data highlights the importance of isolating the right number of intact nuclei, as well as ensuring optimal amplification conditions during library preparation to obtain good‐quality sequence data for downstream analyses. We recommend using fresh tissue samples if possible because we show that direct cryopreservation of the tissue may affect chromatin integrity. This effect could be avoided or reduced by preserving the homogenate in cell culture medium. Overall, we explain the ATAC‐seq protocol and downstream analyses in detail and give step‐by‐step advice to researchers who are new to the field and want to implement this method. With careful planning and validation, ATAC‐seq can reveal the regulatory landscape of a genome and aid in identifying elements that govern gene expression.

## Graphical abstract

Principle of ATAC‐seq (left), overview of arthropod species with published ATAC‐seq data (middle), summary of experimental design (right).

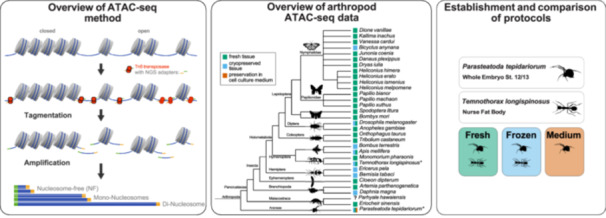

## 1 Introduction

The advent and rapid advancement of DNA sequencing technologies have driven the establishment of genomic and transcriptomic resources for both model and non‐model organisms. While large research communities focus on generating these resources for well‐established model organisms, such as the vinegar fly *Drosophila melanogaster*, the nematode *Caenorhabditis elegans*, or the house mouse, *Mus musculus*, many questions in ecology and evolution research require the study of non‐model or emerging model systems. As a result, the number of available genomes and expression datasets has steadily increased over the years. In particular, long‐read DNA sequencing methods, such as PacBio (Rhoads and Au 2015) and Oxford Nanopore (Wang et al. 2021), in combination with sequencing‐based scaffolding approaches (e.g., HiC, Belton et al. 2012), enable the generation of highly contiguous genome data, even at the chromosome level, for many organisms. Recent large‐scale genome initiatives, such as the Darwin Tree of Life (Darwin Tree of Life Project Consortium 2022) and ERGA (Mc Cartney et al. 2024), which employ these technologies, have contributed to the generation of numerous high‐quality genomes. By integrating multiple lines of evidence, such as gene expression (i.e., transcriptome) data, genomic resources can be structurally annotated to predict protein‐coding regions (Gabriel et al. 2024). The availability of such annotated genomes has facilitated comprehensive comparative genomic and transcriptomic studies across a wide range of emerging model species. However, for most multicellular organisms, only a relatively small fraction of the genome encodes proteins (Sana et al. 2012; Piovesan et al. 2019). The remainder consists of noncoding regions, some of which are important for gene regulation.

Context‐specific gene regulation is essential for proper development and function, as genes must be expressed in the correct cells at the correct time (reviewed, e.g. in Buchberger et al. 2019). Due to the complexity and modularity of regulatory mechanisms, gene expression can be temporally and spatially fine‐tuned (Carroll 2000, 2008; Wray 2007; Wittkopp and Kalay 2011) allowing natural selection to act on this variation without inducing pleiotropic effects (reviewed in Buchberger et al. 2019; Hill et al. 2021). Indeed, natural variation in gene regulation and gene expression is extensive and is thought to play a crucial role in adaptation, as it underlies much of the phenotypic variation within and among species (King and Wilson 1975; Wray 2003, 2007; Wittkopp and Kalay 2011). Thus, to gain mechanistic insights into the function and evolution of organisms it is important to study noncoding genomic regions.

In recent years, several DNA sequencing‐based functional genomics methods have been established (reviewed in Ma and Zhang 2020) to study the interplay between genome and chromatin organization (e.g., HiC, Belton et al. 2012), epigenetic modifications (e.g., EM‐seq, Vaisvila et al. 2021), gene regulation (e.g., ChIP‐seq, Kharchenko et al. 2008; Cut&Run, Hainer and Fazzio 2019; Cut&Tag, Kaya‐Okur et al. 2019), and gene expression (RNA‐seq, Wang et al. 2009). Many of these methods require high‐quality antibodies to detect specific proteins of interest (e.g., transcription factors or specific histone modifications) or have specific requirements for the amount and quality of the starting material. In contrast, Assay for Transposase‐Accessible Chromatin with sequencing (ATAC‐seq) requires significantly less input material, while still allowing to identify putative regulatory regions on a broader scale (Buenrostro et al. 2013; Buenrostro, Wu, Chang, et al. 2015). In this article, we outline the basic principle and major applications of this method. We discuss challenges in establishing ATAC‐seq for emerging model organisms and provide an overview of protocols and benchmarking steps, covering the entire workflow from sample preparation and preservation to data analysis and quality control. We provide case examples for the common house spider, *Parasteatoda tepidariorum*, and the acorn ant, *Temnothorax longispinosus*, for which we performed a systematic comparison of the effects of tissue preservation on ATAC‐seq data quality. With our overview of the method and specific recommendations, we aim to offer a reference for researchers establishing ATAC‐seq protocols in emerging model organisms.

## 2 ATAC‐seq – Applications

As DNA is typically highly condensed to fit within the nucleus, most regulatory DNA regions are tightly wound around nucleosome proteins, rendering them inaccessible to DNA‐binding proteins, such as transcription factors. Protein‐coding genes located in such densely packed heterochromatic domains are usually not transcribed. Active transcription, on the other hand, requires more loosely packed euchromatic domains where the DNA is more accessible to regulatory proteins. Research in model organisms has demonstrated that the distribution of accessible chromatin, and thus transcriptional activity, is specific to a given time point and cell type (Schulze and Wallrath 2007; Burton and Torres‐Padilla 2014). Therefore, identifying accessible chromatin regions at different developmental stages, under varying physiological conditions, or in various tissues holds great potential for gaining novel insights into putative regulatory genomic regions (Klemm et al. 2019). ATAC‐seq can be employed to obtain a comprehensive view of the regulatory landscape of a genome. This method allows for the identification of putative regulatory elements, such as enhancers, promoters, and other regulatory regions that control gene expression. ATAC‐seq provides indirect insights into how transcription factors and other proteins, such as nucleosomes, interact with DNA (Li et al. 2019; Bentsen et al. 2020; Xu et al. 2022). Moreover, it can reveal changes in chromatin structure in response to external stimuli, during development and evolution, or during disease progression (Corces et al. 2016; Davidson and Moczek 2024). Since the gene regulatory landscape is highly context‐specific, single‐cell based approaches to ATAC‐seq enable even the characterization of cell types based on their unique chromatin accessibility patterns (Satpathy et al. 2018; Wu et al. 2022).

**Figure 1:**
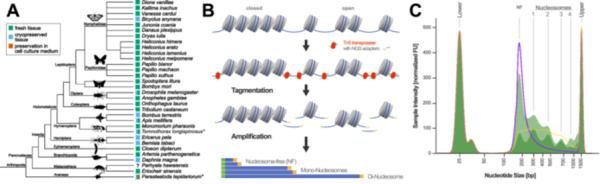
(A) Phylogeny of species with published ATAC‐seq studies, based on Supporting Information S5: Table S1. The boxes at the tips of the tree indicate the type of sample preparation used (green = fresh material, blue = cryopreserved material, orange = preservation in cell culture medium). Data for the two species labeled with asterisks are generated in this study. The animal silhouettes were downloaded from http://phylopic.org/, with credits to Mathilde Cordellier for the spider *Parasteatoda tepidariorum* (https://creativecommons.org/licenses/by-nc/3.0/), Thomas Hegna for *Artemia salina* (https://creativecommons.org/publicdomain/mark/1.0/), T. Michael Keesey for the malacostraca example (https://creativecommons.org/licenses/by-sa/3.0/), Guillaume Dera for *Cloeon dipterum*, T. Michael Keesey for the ant, Lubna Maherally for the honey bee, Max Farnworth for *Tribolium castaneum*, Andy Wilson for the *Drosophila melanogaster*, *Oncopeltus fasciatus* as example for Hemiptera and *Danaus plexippus*, Gemma Martínez‐Redondo for *Bombyx mori*, Mattia Menchetti for *Papilio machaon* (https://creativecommons.org/publicdomain/zero/1.0/). (B) Overview of the ATAC‐seq method, illustrating the key steps that lead to nucleotide fragments of different size classes. Note that the length of the nucleotide sequencing adapters (shown in green and yellow) is downscaled for clarity. (C) Schematic of example fragment size distributions for ATAC‐seq libraries, as determined by instruments such as Agilent Bioanalyzer or Tapestation. The range of acceptable fragment sizes is indicated by the green‐shaded areas. The purple line shows the fragment size distribution of an over‐tagmented library, while the yellow dotted line illustrates the fragment size distribution of an under‐tagmented library. Red dotted lines indicate the lower and upper fragment markers. NF = nucleosome‐free fraction.

Arthropoda are a hyperdiverse phylum including the most species‐rich classes of higher eukaryotes (Zhang 2011; Basset et al. 2012). They have successfully adapted to a wide range of environments, exhibiting numerous phenotypic innovations and adaptations (for some examples, see Villani et al. 1999; Marx et al. 2012; Senji Laxme et al. 2019). Therefore, arthropods serve as excellent models for investigating the genomic and regulatory basis of a variety of traits. ATAC‐seq has become an essential tool for investigating gene regulation and chromatin dynamics in emerging model arthropods (Figure 1A; see Supporting Information S5: Table S1 for a comprehensive list). For example, ATAC‐seq has been successfully applied to study developmental processes in crustaceans (Sun et al. 2022; Hao et al. 2024; Li et al. 2024), spiders (Iwasaki‐Yokozawa et al. 2022), and insects, such as beetles (Mau et al. 2023) and mayflies (Pallarès‐Albanell et al. 2024). Holometabolous insects, which undergo metamorphosis, represent particularly important models for studying developmental biology. The application of ATAC‐seq before, during, and after pupation has helped to identify the dynamics of the regulatory landscape during these transitions, as exemplified in papilionid butterflies (Wan et al. 2022). In social insects, such as bees and ants, ATAC‐seq has been applied to study the regulatory mechanisms underlying caste determination (Wang et al. 2020; Lowe et al. 2022; Zhang et al. 2023) and behavioral plasticity (Jones et al. 2020; Fang et al. 2022).

Insects also serve as important models for deciphering the regulatory mechanisms underlying morphological diversification (Glastad et al. 2019). The application of ATAC‐seq facilitated the identification of genes associated with differences in cuticle color (Wan et al. 2021), as well as the identification of *cis*‐regulatory elements and regulatory networks involved in the evolution of colour patterns, such as eyespots and other wing‐related traits (Mazo‐Vargas et al. 2022; Murugesan et al. 2022; Murugesan and Monteiro 2023; Van Belleghem et al. 2023; Davidson and Moczek 2024; Tendolkar et al. 2024; Zhou et al. 2024) in butterflies. Specifically, chromatin accessibility analysis identified five *cis*‐regulatory elements in the *optix* gene locus that underlie colour pattern variation in *Heliconius* butterflies (Lewis et al. 2019), while transcription factor activity was shown to drive changes in chromatin accessibility during wing metamorphosis in the butterfly *Junonia coenia* (van der Burg et al. 2020). Moreover, structural genomic variants have been linked to the evolution of chromatin accessibility in closely related *Heliconius* butterflies (Ruggieri et al. 2022).

ATAC‐seq has furthermore successfully been applied to understand response to environmental changes. For instance, in the whitefly *Bemisia tabaci*, metabolic responses to temperature stress have been linked to changes in chromatin accessibility (Shen et al. 2023), while in cell cultures of the domestic silk moth *Bombyx mori* changes in chromatin profiles have been observed during viral infections (Kong et al. 2020; Zhao et al. 2023) and in response to hyperproteinemia (Wang et al. 2024).

Overall, the application of ATAC‐seq in emerging model arthropods is rapidly expanding, providing crucial, novel insights into developmental biology, evolutionary processes, and the interaction between genetic and environmental factors.

## 3 ATAC‐seq – A Brief Overview

ATAC‐seq begins with the isolation of cell nuclei and utilizes a hyperactive Tn5 transposase, which preferentially binds to accessible nuclear DNA. The transposase is typically pre‐loaded with synthetic oligonucleotide sequences that can serve as sequencing adapters for Illumina short‐read sequencing. The transposase cleaves accessible DNA and inserts the sequencing adapters (i.e., tagmentation) (Li et al. 2020), upon which the resulting tagged fragments are amplified by PCR and subjected to short‐read DNA sequencing (Buenrostro et al. 2013; Buenrostro, Wu, Chang, et al. 2015; Yan et al. 2020) (Figure 1B). Since transpositions are more likely in open chromatin regions, reads originating from accessible genomic regions will be enriched. These reads are then aligned (i.e., mapped) to a reference genome, and genomic regions with high read counts, known as peaks, are identified as containing relevant regulatory information (Buenrostro et al. 2013; Buenrostro, Wu, Chang, et al. 2015; Yan et al. 2020) (Figure 1B).

Methods that rely on mechanical (FAIRE‐seq, Giresi et al. 2007) or enzymatic (DNAse‐seq, Boyle et al. 2008) fragmentation of accessible genomic DNA follow complex protocols and require a significant amount of starting material (e.g., up to 4 mg of yeast cells for FAIRE‐seq, (Segorbe et al. 2018)). In contrast, ATAC‐seq can be performed with as little as 50,000 cells as starting material (Buenrostro et al. 2013; Buenrostro, Wu, Chang, et al. 2015; Grandi et al. 2022), and even applications that assess chromatin accessibility at the single‐cell level have been established (sc‐ATAC‐seq, Buenrostro, Wu, Litzenburger, et al. 2015). Therefore, ATAC‐seq is particularly well‐suited for studying arthropods, as many species are small, making the collection of larger quantities of biological material difficult. The original ATAC‐seq protocol (first protocol: Buenrostro, Wu, Chang, et al. 2015; improved protocol: Corces et al. 2017) is highly reproducible, relying on fewer steps. This protocol has worked well in mammals (Liu et al. 2019) and has become a standard method when analyzing cell lines (Zhao et al. 2020). However, the protocol has not been as straightforward for other sample types, requiring modifications for different tissues and species (Kissane et al. 2021; Grandi et al. 2022; Zhang et al. 2024). For instance, tissue preservation methods (Figure 1A), homogenization protocols and nuclei isolation procedures must be adjusted for different tissue samples (Fujiwara et al. 2019; Zu et al. 2023 and this study). Therefore, it may be necessary to optimize various details of the protocol when adapting the method for emerging model species. It is important to note that the analysis of ATAC‐seq data requires a reference genome, as the generated nucleotide sequences (i.e., reads) are predominantly located in noncoding genomic regions (Klemm et al. 2019; Yan et al. 2020). Moreover, to fully utilize ATAC‐seq data, it is recommended to integrate complementary functional genomics data (e.g., ChIP‐seq, Cut&Run, Cut&Tag, and methylation profiles) because the method cannot provide specific information about protein‐DNA interactions, such as transcription factor binding. Additionally, integrating ATAC‐seq and gene expression data holds significant potential for linking accessible chromatin regions with transcriptional activity.

## 4 Establishing ATAC‐seq for Emerging Model Arthropods – Laboratory Steps

**Figure 2:**
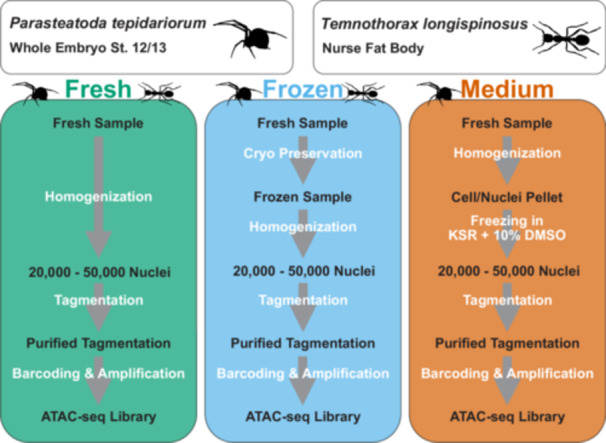
Schematic representation of samples used in this study and the comparison of sample processing methods using either no preservation method (in the following referred to as fresh samples, green box), cryopreservation (in the following referred to as frozen sample, blue box) or cell culture medium based cryopreservation after homogenization (in the following referred to as medium sample, right box). For the ant *Temnothorax longispinosus*, nurse fat body tissue was used to compare a single fresh and a single cryopreserved sample. For the spider *Parasteatoda tepidariorum*, all three methods were compared with 3–4 replicates of whole embryos from later embryonic stages during germ band retraction and dorsal closure (Stage 12/13 according to Mittmann and Wolff 2012).

In the following, we highlight the most critical steps in the ATAC‐seq protocol that, based on our experience, have the highest impact on data quality when optimizing the method for emerging model arthropods (Table 1). We strongly recommend familiarizing oneself with the two most widely accepted protocols (Buenrostro et al. 2013; Buenrostro, Wu, Chang, et al. 2015; Corces et al. 2017), as we will not provide a detailed description of every step. Instead, we provide a protocol used for spider and ant samples in Section 8, and a step‐by‐step ATAC‐seq protocol is available online (https://doi.org/10.25625/NFVW5W). Note that preservation of tissue samples may be required prior to the laboratory steps outlined below. Since most arthropod ATAC‐seq data is based on fresh material (Figure 1A) and a thorough assessment of different tissue preservation methods for ATAC‐seq has not been published to date, we will present and discuss such a comparison in a dedicated section at the end of this study (Figure 2).

**Table 1:** Overview of key steps during the library preparation that we recommend optimizing if ATAC‐seq is established for the first time in an emerging model arthropod system or in a new tissue.

	Important steps	Recommendations and possible modifications	Quality check
Planning phase	Replicates	2–3 technical replicates 3–5 biological replicates	See bioinformatic QC analysis (Table 2)
	Tissue preservation	Optimal: Fresh material Preferred: Nuclei preservation (freezing in cell culture medium) Possible: Flash frozen	
Tissue homogenization	Mechanical	Equipment: For example, dounce homogenizer, TissueRuptor, TissueLyser Number of strokes during homogenization	Individual cells No large debris
	Chemical	Enzyme/detergent concentration Adjust incubation time	
Nuclei extraction	Chemical and mechanical	Detergent concentration Filter through Cell Strainer Centrifugation at 500–1000×*g*	Nuclei number (≥ 50,000)^a^ no debris (see Supporting Information S1: Figure S1)
Tagmentation	Time	30 min (±15 min)	Fragment size distribution (see Figure 1C)
	Concentration	Follow instructions of Tn5 company	
	Purification	Column based	
Library amplification	Initial amplification & barcoding	5 min initial extension at 72°C 5 cycles initial amplification	30–600 ng library DNA (consultation with sequencing facility) Fragment size distribution (see Figure 1C)
	Assess library concentration	Optimal: Real‐time qPCR (same PCR conditions as initial and additional PCR) Possible: Qubit	
	Additional PCR cycles	Based on quantification: 3–12 (total number of cycles: 8–18)	
	Library purification	Column based purification Size selection with beads	
Sequencing	Read length	Optimal: 50–75 bp Possible: 150 bp	See bioinformatic QC analysis (Table 2)
	Read type	Paired‐end reads	
	Number of unique reads^b^	Peak calling: 20–30 million TF footprinting: 100–200 million	

*Note:* This table does not provide a comprehensive overview of all steps but should be used in combination with the recommended standard protocols (Buenrostro et al. 2013; Buenrostro, Wu, Chang, et al. 2015; Corces et al. 2017).

Abbreviation: QC, quality control.

a 50,000 nuclei recommended for human samples; for smaller genomes, more nuclei are recommended (see text).

b Based on 180 Mbp genome of *Drosophila melanogaster*.

### 4.1 Tissue Homogenization and Tagmentation

Since cell lines are rarely established for emerging model organisms, the first crucial step is typically the homogenization of tissue samples to isolate intact nuclei without debris contamination. For efficient tagmentation, the nuclei must be accessible for the Tn5 transposase. Reduced tagmentation efficiency can result from cell/nuclei clumping, inefficient cell homogenization leaving debris (black arrow, Supporting Information S1: Figure S1C), or damage of the nuclei through rupturing or complete lysis (red arrow, Supporting Information S1: Figure S1A) (Adey 2021). We recommend using Dounce homogenizers or pestles for embryos and soft tissues, such as fat bodies, while cell disruptors should be employed for more rigid tissues from adults. Filtration through a cell strainer can help remove unlysed cell clumps and debris. Washing steps can be adjusted to either improve debris removal or minimize the loss of nuclei. Additionally, centrifugation speeds during washing steps can be slightly modified to increase the nuclei yield. It is generally suggested to include detergents, such as NP40 or digitonin, during homogenization, and Tween20 during washing steps (Buenrostro et al. 2013; Buenrostro, Wu, Chang, et al. 2015; Corces et al. 2017), to improve membrane permeability across various cell types, enhance library complexity, and prevent mitochondrial contamination. However, we strongly recommend adjusting the homogenization method and detergent concentration for each tissue type and species to achieve an optimal balance between lysis and nuclei accessibility.

After nuclei isolation, it is important to evaluate both the quality and quantity of nuclei, as the transposase‐to‐nucleus ratio plays a crucial role in achieving efficient DNA fragmentation. While automated cell counters may offer convenience, we have observed inaccuracies, such as counting debris as nuclei, which can skew results. Therefore, we recommend staining the nuclei with either nonfluorescent (e.g., Trypan blue) or fluorescent (e.g., DAPI) dyes to assess their quality (i.e., size and roundness) and to count them using classical microscopy (e.g., Neubauer counting chambers). Optimal samples should consist of minimal debris and mostly intact, individual nuclei (green arrow, Supporting Information S1: Figure S1B). A typical recommendation for human samples is to use 50,000 intact nuclei (Buenrostro et al. 2013; Buenrostro, Wu, Chang, et al. 2015; Corces et al. 2017), although good results can be obtained with 20,000 nuclei (personal communication Diagenode). Note that the method also works with as few as 500 nuclei, but we do not recommend this as lower nuclei input results in reduced sensitivity for peak detection due to high background noise (Buenrostro et al. 2013). Generally, using too few nuclei can lead to an over‐tagmentation, resulting in an overrepresentation of short fragments and a loss of larger nucleosome‐bound DNA fragments (Gehrke and Srivastava 2022) (Figure 1C). Conversely, under‐tagmentation, which can occur when using too many nuclei, could result in an underrepresentation of nucleosome‐free fragments and many long fragments that are less efficiently sequenced (Buenrostro, Wu, Chang, et al. 2015) (Figure 1C). It is important to note that optimal tagmentation depends on the Tn5‐to‐DNA ratio. Therefore, the number nuclei should be increased for small genomes and decreased for large genomes. The recommended 50,000 nuclei for the 3.2 Gbp human genomes can serve as reference for such adjustments. For instance, about 400,000 nuclei were used for ATAC‐seq in *Heliconius* butterflies because their genome is only 454 Mbp genome (Lewis et al. 2019). Instead of optimizing the number of nuclei, the amount of transposase can be adjusted to the number of nuclei to avoid Tn5 oversaturation (Zhang et al. 2021) and tagmentation efficiency should be optimized by adjusting the buffer composition (Xin et al. 2020) or incubation time through a titration experiment (Kissane et al. 2021).

### 4.2 Library Amplification

**Figure 3:**
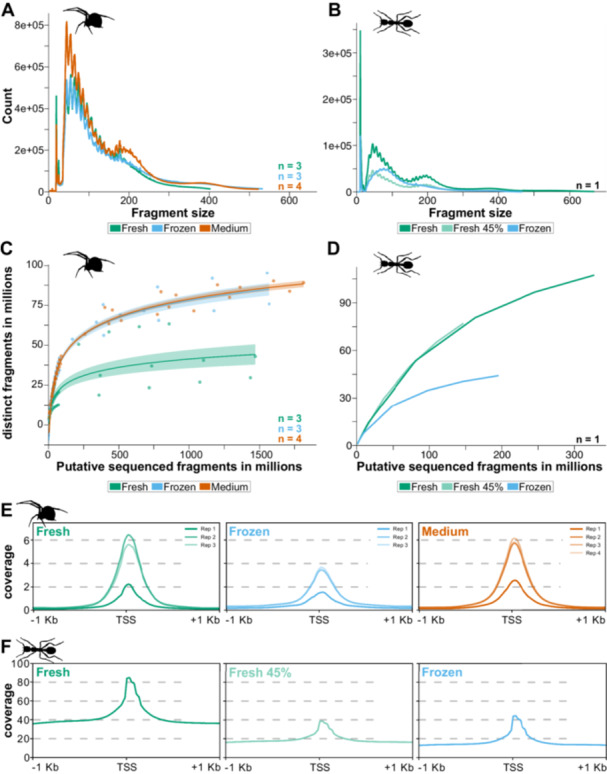
Post‐mapping quality assessment of ATAC‐seq samples. (A) Fragment size distribution of the spider samples using different preservation methods: fresh (green), frozen (blue), medium (orange). Mean values are shown for the replicates. Individual replicate fragment size distributions can be found in Supporting Information S2: Figure S2A. (B) Fragment size distribution of the ant samples for fresh (dark green), fresh subsampled (light green), and frozen (blue) preservation methods. (C) Library complexity estimation for the spider samples using different preservation methods: fresh (green), frozen (blue), medium (orange). Mean values (lines) and standard deviation (transparent shading) are shown for the replicates. Dotted lines show the distinct replicates, and individual replicate fragment size distributions can be found in Supporting Information S2: Figure S2B. (D) Library complexity estimation of the ant samples for fresh (dark green), fresh subsampled (light green), and frozen (blue) preservation method. (E and F) Transcription start site (TSS) enrichment plots for the nucleosome free fraction of the spider replicates (E) and ant (F) samples. Note that the data is only comparable within species and not between species.

After tagmentation, the DNA fragments are barcoded and amplified for Illumina sequencing. The quality and success of library amplification are highly dependent on factors such as the amount of input DNA, the length distribution of fragments (which reflects tagmentation efficiency), and the GC content of the sample (Van den Berge et al. 2022; Rochette et al. 2023). Excessive PCR cycles can lead to overamplification, disproportionally increasing the representation of small DNA fragments and resulting in low‐complexity libraries. Conversely, an insufficient number of PCR cycles may produce inadequate output, especially when starting material is limited. To optimize the number of PCR cycles, we recommend quantitative real‐time PCR (qPCR) (Buenrostro, Wu, Chang, et al. 2015; Kissane et al. 2021), although alternative quantification methods, such as Qubit, can also be used (Grandi et al. 2022). Typically, qPCR is performed on an aliquot of an initial five‐cycle library amplification reaction to determine the number of additional cycles required. The total number of cycles depends on tissue type, species, quality of nuclei isolation, and tagmentation efficiency. Ideally, the total number of cycles should range between 8 and 18, with a maximum of 21 cycles, to ensure amplification stops before saturation (Augusto et al. 2019, 2021) and to avoid overamplification. After library purification, its quality should be assessed using fragment analysis (Figure 1C). If the library contains an excess of large fragments (> 1500 bp), or primer/adapter dimers (< 100 bp), a double‐sided bead‐based size selection should be performed (Zhang et al. 2022). This step helps to eliminate unwanted fragments that can negatively affect sequencing quality. It is crucial to retain fragments between 100 and 1500 bp, as these would include nucleosome‐free regions (~200 bp), as well as mono‐ (~300 bp), di‐ (~500 bp), tri‐nucleosome (~700 bp) fractions. The presence of these different fragment sizes indicates good library quality (Figure 1C). It is important to note that the fragment sizes assessed at this point of the protocol include ~100 bp of tags and sequencing adapters. Therefore, the fragment size distribution before sequencing should not be confused with the one obtained after read mapping during the bioinformatic analysis (see below) where each fraction is shifted by ~100 bp towards smaller fragment sizes (Figure 3A,B).

### 4.3 Number of Replicates and Sequencing

The next critical decisions involve determining the number of replicates, as well as the type and number of sequencing reads (Table 1). For detailed recommendations, we strongly advise consulting the ENCODE guidelines (https://www.encodeproject.org/atac-seq/). Generally, generating at least three biological and two technical replicates is recommended for robust omics datasets. However, in our experience, ATAC‐seq data often exhibits high correlation between replicates, which may allow for omitting technical replicates in favor of including more biological replicates. The decision on replication should also align with the research question. If the goal is a comprehensive quantitative comparison of chromatin accessibility across conditions, such as experimental treatments, developmental stages, or populations, proper replication is essential. Conversely, if the primary aim is an initial exploration of putative regulatory regions for a specific tissue, fewer replicates may suffice.

As the nucleosome‐free fraction of ATAC‐seq libraries is typically below 100 bp, generating reads longer than 75 bp is not advisable. We recommend 50–75 bp reads in paired‐end mode, as mapping read pairs is more efficient and accurate. For standard chromatin accessibility studies, we suggest sequencing at least 50 million unique reads per sample. However, for transcription factor footprinting a higher depth of 200 million reads or more may be necessary, depending on the genome size (Buenrostro et al. 2013; Buenrostro, Wu, Chang, et al. 2015). Optimizations of the standard ATAC‐seq protocol have reduced the number of required reads in some cases by decreasing the fraction of uninformative mitochondrial reads (Corces et al. 2017).

## 5 Establishing ATAC‐seq for Emerging Model Arthropods – Bioinformatics

When adapting the ATAC‐seq method for emerging model organisms, it is crucial to carefully evaluate how modifications in the laboratory protocols affect the resulting data. Below, we present an overview of the most widely used tools and workflows, along with recommendations for steps that require optimization when implementing ATAC‐seq (Table 2). We also highlight critical quality control measures necessary to ensure that the data accurately reflect the biological context of chromatin accessibility, rather than experimental or analytical artifacts (Table 2). These quality control steps are particularly important for validating the reliability of results. We emphasize that this is not an exhaustive review. For a more in‐depth exploration, we encourage readers to consult the following resources: the ENCODE ATAC‐seq guidelines (https://www.encodeproject.org/atac-seq/), the Galaxy ATAC‐seq tutorial (https://training.galaxyproject.org/training-material/topics/epigenetics/tutorials/atac-seq/tutorial.html), and a comprehensive review on ATAC‐seq methodology (Yan et al. 2020).

**Table 2:** List of the most important quality metrics with which ATAC‐seq data should comply, and how to obtain them.

	Approach	Tool	Optimum
Raw read QC	Raw read QC	FastQC/MultiQC	Follow FastQC recommendations
	Trimming	Trimmomatic/TrimGalore	No adapter leftovers remain Removal of low‐quality nucleotide calls
	*Option1*: mt‐contamination removal	BlastN	< 1%–5% mt sequences in the dataset
Post‐mapping QC	Mapping rates	Qualimap	Alignment rate > 95% (80% acceptable)^a^ ≥ 25 million single‐, or ≥ 50 million paired‐end nonduplicate, non‐mt reads^a^
	*Option2:* mt‐contamination removal	Bamtools (*‐split*)	< 1%–5% mt sequences in the dataset
	Library complexity	ATACseqQC/Picard	Curve reaches saturation
	Duplicate removal	Picard	No duplicates left
	Fragment size distribution	ATACseqQC/Picard	Should follow expected distribution (see Figure 3A,B)
	Bam coverage normalization	Samtools (*‐view*)	Comparable coverage across samples/replicates
	Correlation across replicates	Deeptools (*‐multiBamSummary*)	> 70% of coverage correlation between replicates
Post‐peak QC	FRiP score (fraction of reads in peaks)	Deeptools	≥ 30%^a^
	Peaks per replicate	Peak caller output	> 150,000 (> 100,000 acceptable)^a^ (but may depend on age/tissue/developmental stage)
	*q*‐value distribution	For example, MACS	Lower *q*‐values indicate higher confidence
	Peak overlap across peak callers/replicates	Bedtools (*‐intersect*)	Keep peaks that show an overlap between two different peak calling algorithms or/and between replicates

*Note:* Asterisks indicate values based on the current ENCODE standards (https://www.encodeproject.org/atac-seq/). Note that this table does not provide a detailed overview of bioinformatic analysis steps.

Abbreviations: mt, mitochondrial; QC, quality control.

a Note that signal‐to‐noise estimates, such as the FRiP score, may be influenced by genome size (see text for details).

### 5.1 Raw Read Quality Control

The first step in any ATAC‐seq analysis should involve performing raw read quality control, using tools such as FastQC (https://www.bioinformatics.babraham.ac.uk/projects/fastqc/). This should be followed by adapter trimming with software such as Trimmomatic (Bolger et al. 2014) or TrimGalore (https://github.com/FelixKrueger/TrimGalore). For datasets based on reads longer than 75 bp, high levels of adapter contamination are expected due to the short fragments derived from the nucleosome‐free fraction. Although trimming may result in a substantial reduction of the data quantity, this step must be carefully executed to ensure accurate read mapping (discussed below). If significant PCR amplification of the library was required (see above), the FastQC report may reveal a high level of sequence duplication. While some duplicated reads may originate from sequencing adapters, they are more often the result of PCR duplicates. We recommend removing these duplicate reads after mapping (see below).

Another crucial step is removing mitochondrial read contamination. Mitochondrial reads can be identified by including the mitochondrial genome during mapping (see below) or by searching all reads against the mitochondrial genome using the software BlastN (Altschul et al. 1990), or the Magic‐Blast tool specifically designed to map RNA‐reads (Boratyn et al. 2019). Optimal removal of mitochondrial reads is most efficient when a species‐specific mitochondrial genome is available. For emerging model organisms lacking such a reference, the mitochondrial genome of a closely related species may serve as an alternative.

### 5.2 Mapping and Post‐Mapping Quality Control

Reads are mapped to a reference genome using commonly applied mapping tools, such as Bowtie2 (with the *‐‐very‐sensitive* option) (Langmead and Salzberg 2012), HISAT and BWA‐MEM (Li 2013) (https://github.com/lh3/bwa). It is generally recommended to exclude reads derived from fragments larger than 2000 bp, for example, by setting the ‐X option in Bowtie2 to 2000 or by filtering after mapping if BWA‐MEM is used. Mapping quality depends on several factors, including read quality, with paired‐end reads offering higher confidence for the mapping position, and the quality of the genome assembly. As paired‐end reads originating from large fragments may fail to map properly to highly fragmented, scaffold‐level genomes, we strongly recommend using chromosome‐level or high‐quality scaffold‐level assemblies as mapping references. Mapping quality can be assessed and compared across samples using tools such as Qualimap (García‐Alcalde et al. 2012). After mapping, it is advisable to remove duplicate reads using tools such as Picard (https://broadinstitute.github.io/picard) because these reads will artificially affect coverage estimations and thus reduce reproducibility of the ATAC‐seq data (Pranzatelli et al. 2018). Moreover, we recommend removing reads that map to multiple locations in the genome as they may represent repetitive sequences, which could lead to false‐positive peaks. In addition to general post‐mapping quality assessments, ATAC‐seq‐specific tests should also be performed. These include estimating library complexity (*estimateLibComplexity*) and examining fragment size distribution (*fragSizeDist*), which can be accomplished using, for instance, the ATACseqQC software (Ou et al. 2018) (e.g., Figure 3A–D, Table 2). Another analysis to assess the quality of ATAC‐seq data leverages the well‐established observation that nucleosome‐free fragments are predominantly located at transcription start sites (TSSs), while nucleosome‐bound regions typically flank sites of active transcription initiation (Buenrostro et al. 2013). To validate this, the density of reads from short fragments (i.e., nucleosome‐free fraction, < 100 bp) and longer fragments (e.g., mono‐nucleosomal fraction, ~200 bp) can be plotted at TSSs and within 1,000 bp up‐ and downstream regions. This analysis should reveal an enrichment of the nucleosome‐free reads precisely at the TSS (see, e.g., Figure 3E,F), accompanied by a bimodal distribution of mono‐nucleosomal reads flanking the TSS. This expected distribution of nucleosome‐free and mono‐nucleosomal reads also allows drawing conclusions about the integrity of the chromatin at the time of Tn5 tagmentation.

It is worth noting that some protocols (Buenrostro et al. 2013; Corces et al. 2017) include a post‐mapping step to shift the reads. This step accounts for the unique molecular behavior of the Tn5 transposase, which creates a 9‐nucleotide sequence duplication during DNA repair (Adey et al. 2010). This adjustment may only be necessary for downstream analyses requiring single‐nucleotide resolution, such as transcription factor footprinting.

### 5.3 Peak Calling and Post‐Peak Quality Control

After reads are mapped and prior to peak calling, it is essential to normalize library sizes between biological replicates (e.g., using samtools, Li et al. 2009) to avoid variation of peak number and peak quality due to differences in read coverage across replicates. Eventually, peaks are identified employing MACS2 (Zhang et al. 2008), which is the most widely used tool for peak calling. While several other peak callers are available (Ibrahim et al. 2015; Tarbell and Liu 2019; Hentges et al. 2022; Vu et al. 2023) (https://github.com/jsh58/Genrich), to date, no comprehensive comparison or benchmarking of different peak callers specifically for ATAC‐seq data has been published. However, web‐based resources (e.g., https://bigmonty12.github.io/peak-calling-benchmark) may provide useful insights to guide tool selection. We recommend starting with MACS2 and potentially comparing results with other peak callers to ensure consistency and robustness of the analysis. It may be advisable to call peaks using multiple tools and use a consensus peak set based on different peak callers and replicates for further analyses.

**Figure 4:**
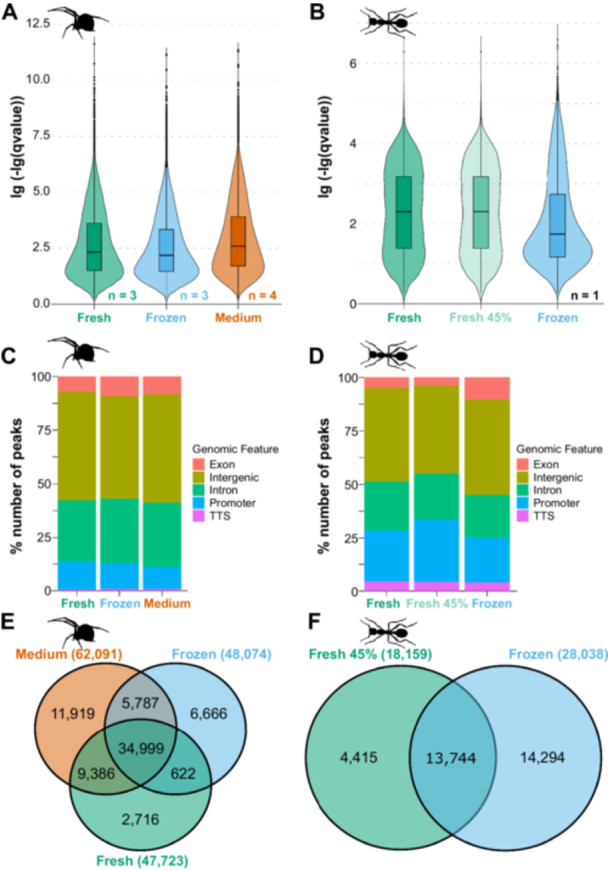
Post‐peak calling quality assessment of ATAC‐seq samples. (A) Comparison of lg(‐lg(*q*‐values)) across treatments for the spider samples. A high lg(‐lg(*q*‐value)) indicates that a peak is highly statistically significant (an FDR of 0.05 corresponds to 1.301 in the plots) and less likely to be a false positive. The cumulative *q*‐values for all replicates are used. Refer to Supporting Information S2: Figure S2C for individual plots for each replicate. All pairwise comparisons were significantly different (Pairwise Wilcoxon Rank Sum Tests; Fresh – Frozen: *W* = 35,503,230,972, *p* < 2e^−16^; Fresh – Medium: *W* = 49,855,901,049, *p* < 2e^−16^; Frozen – Medium: *W* = 43,377,046,641, *p* < 2e^−16^). (B) Comparison of lg(‐lg(*q*‐values)) across treatments for the ant samples. A high lg(‐lg(*q*‐value)) indicates that a peak is highly statistically significant (an FDR of 0.05 corresponds to 1.301 in the plots) and less likely to be a false positive. All pairwise comparisons were significantly different (Pairwise Wilcoxon Rank Sum Tests; Fresh – Frozen: *W* = 290,784,030, *p* < 2.2e^−16^; Fresh 45% – Frozen: *W* = 277,107,730, *p* < 2.2e^−16^; Fresh – Fresh 45%: *W* = 261,656,932, *p* < 2.2e^−16^). Note that higher values for lg(‐lg(*q*‐values) represent high confidence in the called peaks. (C and D) Fraction of peaks located in different genomic regions in (C) spider samples and (D) ant samples. (E and F) Venn diagrams depicting the number of shared and unique peaks across treatments in (E) spider and (F) ant samples are shown. The total number of peaks called per treatment is given in parentheses next to the treatment.

After peak identification, additional quality checks are recommended, including calculating the fraction of total reads in called peaks (FRiP) scores (e.g., using deepTools2, Ramírez et al. 2016), which allow judging the signal‐to‐noise ratio for a given dataset. If, for instance, the chromatin integrity is not preserved during the processing of tissue samples, it is expected to obtain many reads from random genomic regions that do not represent biologically relevant accessible chromatin regions. Accordingly, the overall level of background noise is high, and the FRiP scores are low. Generally, FRiP scores should exceed 0.3 for most samples to meet quality standards (https://www.encodeproject.org/atac-seq/). However, it has been shown that estimates of signal‐to‐noise ratios are influenced by genome size as larger genomes tend to result in more unspecific reads that contribute to background noise (Schmitz et al. 2022). Accordingly, lower FRiP scores may be acceptable for large genomes, while higher scores should be expected for ATAC‐seq data obtained for small genomes. Along the same line, one should assess the *q*‐values provided, for instance, by the MACS2 peak caller, as they represent the minimum false discovery rate at which a significant peak is called (Zhang et al. 2008). Accordingly, lower *q*‐values represent higher confidence in the called peaks (see below, Figure 4A,B; note that *q*‐values are ‐lg‐transformed in this figure and thus higher values represent higher confidence). In addition to the dataset‐wide assessment employing FRiP scores and *q*‐values, the signal‐to‐noise ratio can also be visually assessed for specific genomic regions and gene loci using genome browser tools, such as the Integrative Genomics Viewer (IGV) (Thorvaldsdottir et al. 2013) (see Supporting Information S3: Figure S3 and Supporting Information S4: Figure S4 for representative visualizations).

### 5.4 Downstream Analyses and Data Integration

After peaks are successfully called, the next step is to annotate them to the nearest gene locus to identify which accessible chromatin regions may be linked to specific genes. Tools such as HOMER (Heinz et al. 2010) facilitate peak‐to‐gene annotation and generate summary statistics on peak distribution across various genomic features, such as promotors, untranslated regions, introns, exons, and intergenic regions. These summaries also serve as a quality control measure, as ATAC‐seq reads are expected to be predominantly located in putative regulatory regions, with enrichment in promotors, intergenic regions, and introns (Yan et al. 2020) (see below, Figure 4C,D). For example, if most peaks are found in exons, this could indicate loss of chromatin integrity during sample preparation, which leads to Tn5 transposition in biologically irrelevant genomic region and thus overall low‐quality ATAC‐seq data. Alternatively, inaccurate peak‐to‐gene annotations may result from incomplete or erroneous genome annotations. To avoid such issues, we strongly recommend using high‐quality genome annotations and contiguous genome assemblies for the species of interest. These resources are crucial for the accurate interpretation of ATAC‐seq data.

While peak annotation provides first insights into putative regulatory regions associated with each gene, the true potential of ATAC‐seq emerges when combined with other functional genomics and gene expression data. One of the most accessible approaches, especially for emerging model systems, is integrating ATAC‐seq with RNA‐seq data. This combination facilitates the correlation of putative regulatory regions within a gene locus with the gene's transcriptional activity. For instance, if a gene is expressed in condition 1 but not in condition 2, comparing accessible chromatin between the conditions can help identify regions that are open and closed, potentially highlighting key regulatory elements. Incorporating information about transcription factor binding motifs (e.g., from model system‐derived databases, Matys 2006; Rauluseviciute et al. 2024; Vorontsov et al. 2024) within these regions can further elucidate how a gene of interest is regulated and how it is integrated within the broader gene regulatory network.

Beyond providing mechanistic insights into gene regulation, ATAC‐seq is a powerful tool for comparing regulatory landscapes between species or sexes. For example, it has been used to study differences in open chromatin in human and chimpanzee neural crest cells (Prescott et al. 2015), regulatory variation between *Drosophila* species (Khodursky et al. 2023) and sex‐specific chromatin accessibility in crows (Catalán et al. 2023). Such comparative analyses are well‐suited for emerging model systems with unique phenotypic traits or ecological specializations. However, the success of these systems depends heavily on a comparable quality of the ATAC‐seq libraries, the quality of the genome assembly and annotation for the species involved, as well as their phylogenetic relationship. When comparing closely related species with well‐annotated genomes (Khodursky et al. 2023), ATAC‐seq reads from both species can be cross‐mapped to both genomes to minimize biases in mapping quality. While genome assembly quality has been steadily improving for emerging model systems, annotation quality has not always kept pace (for recent advances in this area, refer to: https://www.ncbi.nlm.nih.gov/refseq/annotation_euk/process/). Furthermore, a close phylogenetic relationship facilitates the accurate assignment of orthologous genes, which is essential for meaningful interpretation of comparative ATAC‐seq data. In contrast, as the evolutionary distance between species increases, the ability to assign clear orthologous relationships diminishes (Natsidis et al. 2021). This poses a significant limitation for applying ATAC‐seq across broad phylogenetic frameworks, as the number of comparable genes decreases with increasing evolutionary distance. However, if the research focuses on a specific subset of genes with clear orthology across species and is supported by high‐quality genome assemblies and annotations, ATAC‐seq remains a powerful method for uncovering mechanistic insights into evolutionary biology.

## 6 Comparison of Preservation Methods

Besides the challenges during the generation and analysis of ATAC‐seq data for emerging model organisms outlined above, it might also be challenging to directly access and process enough fresh material. Especially in study systems with tiny organisms, comparably long generation times, species lacking established laboratory cultures, or species requiring field work collections, it might be necessary to separate sample collection and the ATAC‐seq processing steps by preserving the material in between.

A brief literature survey suggests that flash freezing/cryopreservation of tissue prior to nuclei extraction and library preparation might represent a promising preservation method. The omni‐ATAC‐seq protocol comes with detailed guidelines and protocols for cryopreserved sections (Corces et al. 2017, 2019) and different freezing strategies have been compared (Peng et al. 2021). However, the reproducibility and success of ATAC‐seq from flash‐frozen tissue samples seems to depend on the tissue and adjustments are required (Cejas and Long 2022; Nair et al. 2022). While cryopreservation has been extensively assessed for mammalian tissues and cell culture conditions, very few studies have addressed this question for arthropod samples which have additional specific challenges, such as a hardened cuticle that may impact tissue dissociation, the presence of large amounts of yolk during embryogenesis, and limitations regarding sample accessibility due to small body sizes. A successful implementation of ATAC‐seq after cryopreservation has been published for the water flea *Daphnia magna* (Kissane et al. 2021), and a preliminary protocol for *D. melanogaster* brains is available (unpublished fork of Corces et al. 2019: https://www.protocols.io/view/simplified-atac-seq-protocol-on-frozen-brains-of-f-n92ld8b8ov5b/v1). However, to the best of our knowledge, a systematic comparison of any tissue preservation method with freshly processed samples is currently not available for arthropods. This is especially important because the use of fresh material, where chromatin conformations are optimally represented, are considered the golden standard for ATAC‐seq. Therefore, we compared two freezing preservation methods for embryos of the common house spider *P. tepidariorum* (Figure 2).

The first preservation approach was flash freezing the tissue in liquid nitrogen and subsequent storage at −70°C (Figure 2, center). The second preservation approach included the homogenization of fresh tissue prior to long‐term storage at −70°C in cell culture medium Knock‐out Serum Replacement and 10% DMSO (Figure 2, right). In addition to the spider data, we compared ATAC‐seq data generated from fresh and flash frozen fat bodies from the ant *T. longispinosus* (Figure 2). For a detailed procedure, refer to Section 8 and a step‐by‐step protocol for arthropods (https://doi.org/10.25625/NFVW5W).

For the comparison of preservation methods, we performed the key quality controls outlined above. After nuclei isolation, we performed tagmentation for up to 50,000 nuclei and libraries were amplified using 10–16 PCR cycles depending on the qPCR results (Table 3; Supporting Information S6: Table S2). To evaluate successful tagmentation and library amplification, all libraries were checked for the typical nucleosome fragment size distribution using Bioanalyzer or Tapestation (see Figure 1C for an example). For libraries with repeated nucleosome patterns and no primer contamination, 50–150 bp paired‐end reads were sequenced using Illumina. Quality and duplication levels of the raw reads were analyzed, and adapter sequences, duplicates, and low‐quality reads were removed to generate 11–82 million unique paired‐end reads (Table 3; Supporting Information S6: Table S2). An analysis of the number of mitochondrial reads showed low levels of mitochondrial contamination (0.37%–3.24%, Table 3; Supporting Information S6: Table S2), suggesting that the isolation of nuclei and transposition of nuclear DNA was successful.

**Table 3:** Summary statistics for ATAC‐seq samples.

	Spider			Ant		
	Fresh	Frozen	Medium	Fresh	Fresh (45%)	Frozen
Library amplification^a^	16	13–14	13–16	10	NA	16
Read length (bp)	51 and 150 bp	51 and 150 bp	51 and 150 bp	150 bp	150 bp	150 bp
# PE reads	185,294,859 (±24,050,613)	184,068,657 (±61,169,393)	219,818,490 (±27,810,071)	31,746,169	NA	17,063,898
% Alignment rate	92.60 (±6.83)	94.62 (±4.66)	94.63 (±6.66)	85.61	NA	86.41
# Non‐duplicate reads	36,450,459 (±12,863,921)	63,491,996 (±17,668,643)	72,443,615 (±11,275,322)	19,879,965	10,785,931	11,553,643
% Mitochondrial reads	2.75 (±1.88)	0.80 (±0.33)	1.10 (±1.09)	8.40E+08	NA	3.14E+09
# Peaks	89,700 (±38,989)	83,306 (±8,120)	102,641 (±9,216)	24,033	18,159	28,038
# Reads per peak	420.98 (±118,57)	756.01 (±160.08)	708.78 (±114.45)	541	432	584
# Consensus peaks	47,723	48,074	62,091	NA	NA	NA
% FRiP score	56.72 (±13.92)	38.29 (±5.43)	52.86 (±7.82)	35	31	31
Coverage (X)	19.98 (±11.14)	21.12 (±13.86)	25.20 (±11.44)	6	3.2	3.1
Genome size	1.13 Gbp			332 Mbp		

*Note:* For the replicates of spider samples, mean values and standard deviation are provided. Refer to Supporting Information S6: Table S2 for the statistics for each replicate.

a Total number of PCR cycles (5 initial cycles + *x* cycles after library quantification).

High‐quality reads were mapped against the respective reference genomes resulting in 78%–98% mapping rates (Table 3; Supporting Information S6: Table S2). The fragment size distribution after mapping for both species showed clear peaks at 80–100 bp (Figure 3A,B; Supporting Information S2: Figure S2A), suggesting many nucleosome‐free fragments were present in all samples. A direct comparison of the ant samples showed a clear reduction of larger fragments representing the mono‐ (~200 bp) and di‐nucleosome (~400 bp) fraction in the cryopreserved flash frozen sample in comparison to the fresh sample (Figure 3B). This effect may partially be explained by a lower number of sequenced reads as random subsampling (i.e., 45% of the full dataset) of the fresh sample down to a similar number of reads to the frozen sample also resulted in a reduction of large fragments (“Fresh” vs. “Fresh 45%” in Figure 3B). However, overall library complexity was not different between the full and the subsampled fresh dataset, while it was clearly reduced in the flash frozen sample (Figure 3D). For the spider samples, the replicates were extremely variable (Supporting Information S2: Figure S2A,B; see Section 8 for details). While the cell culture medium preservation method resulted in clear periodic fragment sizes representing the nucleosome free fractions as well as mono‐ (~200 bp) and di‐nucleosome (~400 bp) fractions, the larger fragments were reduced in the fresh and flash frozen samples, respectively (Figure 3A; Supporting Information S2: Figure S2A). Library complexity for the flash frozen and medium preserved samples was comparable (Figure 3C); however, two replicates of the fresh samples showed low library complexity (Supporting Information S2: Figure S2B). This reduction in library complexity may partially explain the loss of larger fragments and was most likely caused by over‐amplification (see Section 8 for details). Therefore, we assessed the PCR duplication levels after read mapping (Table 3; Supporting Information S6: Table S2), which indeed revealed higher fragment duplication levels for the fresh (58.0%–86.5%) compared to the frozen and medium preserved samples (frozen: 44.9%–65.8%; medium: 57.4%–67.7%). The duplicated reads were removed using Picard (Table 3; Supporting Information S6: Table S2) (see Table 2).

To evaluate how well the different preservation methods preserve chromatin integrity, we next analyzed the mapping and peak calling quality (summarized in Table 2). As reads originating from short nucleosome‐free fragments should be enriched at TSSs (Buenrostro et al. 2013), we extracted the duplicate‐free mapped read‐pairs of the nucleosome‐free fraction (< 100 bp) and compared their coverage at TSS (Figure 3E,F). The samples from the ant revealed a more pronounced TSS enrichment in the fresh sample compared to the flash frozen sample (Figure 3F). However, after subsampling the fresh sample to match the read numbers, the TSS enrichment was comparable between treatments (Figure 3F), suggesting similar conservation of chromatin integrity for fresh versus flash frozen ant fat body tissue. In contrast, the flash frozen spider samples showed a consistently reduced TSS enrichment compared to the fresh and medium preserved samples (Figure 3E). Therefore, our spider data suggest that chromatin integrity may be affected by flash‐freezing of embryonic tissue.

Peak calling resulted in a total of 18,000–134,000 peaks (Table 3; Supporting Information S6: Table S2), which we used to calculate the fraction of reads in peaks (FRiP score). All our datasets had values above the recommended threshold of 30% (Table 3; Supporting Information S6: Table S2). While the FRiP scores were comparable for fresh and flash frozen ant samples, we observed much higher values for the fresh and medium‐preserved samples compared to the flash frozen samples for the spider samples (Table 3; Supporting Information S6: Table S2). This finding suggests a higher level of background noise in the flash‐frozen spider samples. To further assess the quality of the called peaks between treatments, we compared *q*‐values obtained from MACS2 (Figure 4A,B; Supporting Information S2: Figure S2C) and the location of the peaks with respect to genomic features, such as exons and introns (Figure 4C,D). In both species, flash freezing resulted in the least optimal *q*‐value distribution (Figure 4A,B; Supporting Information S2: Figure S2C), and among the three treatments for spider samples, we observed the best *q*‐values for the medium preservation (Figure 4A; Supporting Information S2: Figure S2C). The lower *q*‐values of the flash frozen ant samples (Figure 4B) could be in line with more peaks in exon regions (Figure 4D), where ATAC‐seq peaks are generally less likely to be enriched (Buenrostro et al. 2013; Yan et al. 2020). However, as we have no replicates, we cannot exclude random effects. Qualitative visual inspection of the ATAC‐seq data showed no clear differences in signal‐to‐noise ratios between preservation methods in spiders (Supporting Information S3: Figure S3) and ants (Supporting Information S4: Figure S4).

As the ATAC‐seq data for the different treatments were obtained from the same tissue in each species, we expected a large overlap between peaks across treatments within a species. Therefore, we asked how many peaks overlapped between the different treatments within each species (Figure 4E,F). In the spider data (Figure 4E; medium: 56%, frozen: 73%, fresh: 73% of all consensus peaks in each treatment) and in the ant data (Figure 4F; fresh: 76%, frozen: 49% of all peaks in each treatment), many peaks were shared across all three treatments, implying an overall high consistency of the data. Peaks that are unique for a certain treatment can be explained by a generally better signal‐to‐noise ratio, which allows for more efficient peak calling (Corces et al. 2017). However, unique peaks could also be called if chromatin integrity is not properly preserved, and random peaks appear in regions of the genome that do not represent biologically relevant accessible DNA. In the ant data, we found that 51% of all peaks were unique (Figure 4F; 42% unique peaks for the full fresh dataset, Supporting Information S2: Figure S2E). In light of other quality measures, such as low *q*‐values (Figure 4B) and a high proportion of peaks in exon sequences (Figure 4D), this observation suggests some level of nonspecific peaks that may not represent biologically relevant accessible chromatin regions in the frozen ant sample. In the spider data, we identified the highest number of unique peaks in the medium preserved samples (Figure 4E, 19% of all consensus peaks in this treatment). As these samples showed the highest *q*‐values (Figure 4A) and high FRiP scores (Table 3; Supporting Information S6: Table S2), we assume that more efficient peak calling may contribute to the increased number of unique peaks. In line with overall lower library complexity of two replicates of fresh samples (see also Section 8), the fresh samples had the lowest number of unique peaks (Figure 4E, 6% of all consensus peaks in this treatment). As we had replicates for each treatment for the spider data, we also analyzed the number of shared and unique peaks across replicates (Supporting Information S2: Figure S2D). Except for one replicate that had 50% unique and 35% shared peaks, the fresh samples had the largest overlap (70%–72%) and the least number of unique peaks (8%–9%). The medium preserved samples had 5%–18% unique peaks and 56%–69% shared peaks, while the frozen samples had 13%–23% unique peaks and 52%–61% shared peaks among replicates (Supporting Information S2: Figure S2D). The integration of replicate information suggests that processing fresh material allows for the most consistent peak calling if the libraries are of comparable quality, although this finding could also be the result of the overall lower library complexity (see Section 8). The medium‐based preservation resulted in slightly more consistent results than flash freezing preservation.

It is important to note that the interpretability of our data would have benefited from replicates for the ant data and lower variability across spider replicates. The latter issue could partially be explained by differences during library preparation (see Section 8), for instance, the assessment of fragment size distribution (Figure 3A, Supporting Information S2: Figure S2A) and library complexity (Figure 3C, Supporting Information S2: Figure S2B) implied low library quality for two of the fresh samples. This may have led to discrepancies in the peak quality controls as the TSS enrichment suggested that fresh samples were superior to both preservation methods (Figure 3E), while the *q*‐values were better for the medium preserved samples (Figure 4A, Supporting Information S2: Figure S2C). Therefore, our data showed that it is crucial to combine quality controls at all levels of the ATAC‐seq protocol and data analysis to evaluate the usability of the generated data.

Overall, comparison of the results from preserved tissue (i.e., flash freezing and medium preservation) to fresh tissue derived data revealed comparable results in most quality controls for both species. However, our assessment of peak quality in the ant data suggests that flash freezing of tissue may influence peak calling. Similarly, the spider TSS enrichment data and peak quality assessment suggest that the chromatin integrity may be affected by flash freezing. Therefore, despite the mixed results for our fresh spider samples, we strongly recommend using fresh material if feasible as this is generally accepted in the field. If this option is impossible due to challenging collection or shipment requirements, we recommend using medium‐based preservation; however, if these two options are unavailable, flash frozen tissue will still lead to meaningful results, despite a potential impact on chromatin integrity and thus reduced quality.

## 7 Conclusion

ATAC‐seq is a versatile and widely used method for identifying accessible chromatin regions within a genome. These regions play a crucial role in gene regulation, as they often correspond to regulatory elements such as promoters and enhancers. ATAC‐seq has been successfully applied to both established and emerging model organisms, including various arthropods (Figure 1A). However, establishing ATAC‐seq for emerging model arthropods presents several challenges. Key issues include the absence of a reference genome, incomplete or poor genome annotations, and the need for meticulous optimization of the ATAC‐seq protocol to suit the specific organism and tissue. Based on the ATAC‐seq data, we generated for ants and spiders, we strongly recommend optimizing the protocol in conjunction with thorough quality controls at multiple steps of the protocol (Table 1) and the bioinformatic analysis (Table 2). Special emphasis during the protocol should be on tissue homogenization and lysis to obtain an optimal number and quality of the isolated nuclei, ideal tagmentation conditions, as well as optimal library quantification and amplification. As our comparison of preservation methods suggested that cryopreservation of tissue samples may interfere with chromatin integrity, fresh material, or dissociated cells or nuclei frozen in cell culture medium should be used for ATAC‐seq if possible.

With careful planning and thorough validation, ATAC‐seq can reveal the regulatory landscape of a genome and identify elements that control gene expression. ATAC‐seq remains a powerful tool for investigating gene regulation in emerging model arthropods. This information can be leveraged to study a wide range of biological processes, including development, evolution, and the response to environmental changes. In light of restricted availability of ATAC‐seq data in arthropods (Figure 1A), the ongoing community‐driven efforts to sequence and annotate genomes will facilitate the application of ATAC‐seq in more arthropod systems, which will ultimately enable us to capture the genetic biodiversity of organisms and understand how it drives phenotypic diversity across species.

## 8 Materials and Methods

### 8.1 ATAC‐seq Data Generation

A step‐by‐step protocol with important steps that should be used to optimize the procedure, as well as details about buffers and reagents are provided online (https://doi.org/10.25625/NFVW5W).

#### 8.1.1 Tissue Homogenization and Nuclei Extraction

Before tagmentation, homogenization of the tissue and extraction of nuclei were performed by using the previously published OMNI ATAC protocol (Corces et al. 2017) (omitting the Iodixanol steps) in combination with the Kaestner Lab protocol (https://www.med.upenn.edu/kaestnerlab/assets/user-content/documents/ATAC-seq-Protocol-(Omni)-Kaestner-Lab.pdf) with minor modifications depending on the species outlined below (see also https://doi.org/10.25625/NFVW5W). For the spider (*P. tepidariorum*) samples, approximately half a cocoon of embryos from a mix of retraction and dorsal closure stages (Stage 12/13 according to Mittmann and Wolff 2012) was used for extraction. After identification of the developmental stage, the untreated whole embryos were homogenized by douncing with pestle A for 10 strokes and pestle B for 20 strokes using a pre‐chilled Dounce homogenizer (2 mL) with 1–2 mL of cold 1× Homogenization Buffer (final NP‐40 concentration increased fivefold in comparison to the OMNI ATAC protocol and 15‐fold compared to the Kaestner lab protocol). In addition, we used a final concentration of 0.025% Digitonin, as a previous publication on *D. melanogaster* pupal wing tissues used higher Digitonin concentrations (Xin et al. 2020). Note that the final NP40 and Digitonin concentration can be adjusted depending on the tissue and species used. For the ant (*T. longispinosus*) samples, dissected fat body tissue of a pool of 10 nurses was transferred to a pre‐cooled Dounce Micro homogenizer (0.1 mL) containing 650 µL of cold 1× Homogenization Buffer. 20 strokes were performed to obtain a cell suspension. The following steps were carried out the same for both spider and ant samples: The homogenate was incubated on ice for 10 min and filtered through a 20 µm pluriStrainer (pluriselect.com). The filtered suspension was centrifuged at 500–1000 RCF for 5 min to pellet the nuclei. The supernatant was removed carefully, and the pellet was washed twice with 1 mL cold ATAC‐RSB with Tween‐20 at a final concentration of 1.0% (final Tween‐20 concentration increased 10‐fold in comparison to the Kaestner lab protocol, as previously used for *D. melanogaster* pupal wing tissue [Xin et al. 2020]), which increased the number of intact nuclei. Please note that depending on the tissue and species, this detergent concentration can be adjusted between 0.1% and 1%. After washing, the pellet was resuspended in 2× TD Buffer.

#### 8.1.2 Nuclei Counting

For the spider replicates (fresh 2, fresh 3, frozen 2, frozen 3, medium 2, medium 3, medium 4), 2 µL of resuspended nuclei were mixed with 16 µL ultrapure water and 2 µL DAPI (1:1000 from a 10 mg/mL stock solution) and counted using a Neubauer improved chamber (0, 100 mm depth) and a Zeiss Axioplan fluorescent microscope. Intact isolated nuclei should have a strong fluorescent signal without any debris surrounding them and appear spherical (Supporting Information S1: Figure 1B). Nuclei concentration was calculated, and 50,000 nuclei were used for tagmentation. For the ant fat body suspension and the spider replicates (fresh 1, frozen 1, medium 1), the Countess II automatic cell counter (Thermo Fisher Scientific) was used. For the ant fat body counting, cells in 10 µL from the suspension resulted in a low count. For this reason, for the ant samples, a total volume of 30 µL was used for the tagmentation step, while for the spider replicates (fresh 1, frozen 1, medium 1) volumes corresponding to 50,000 nuclei were used for tagmentation. Please note that the automatic cell counter did not generate precise results because it did not discriminate between intact and disrupted nuclei or debris. Thus, using a cell chamber and microscope is recommended.

#### 8.1.3 Tagmentation and Library Preparation

The following steps were performed for both spider and ant samples identically. A 50 µL tagmentation reaction was performed on the nuclei using Tn5 transposase (Diagenode) for 30 min at 37°C following the manufacturer's protocol. The reaction was purified using the MinElute PCR purification kit (Qiagen). All libraries were amplified using specified PCR conditions (Buenrostro, Wu, Chang, et al. 2015) and UDI primer pairs from Diagenode. For most spider replicates (fresh 2, fresh 3, frozen 2, frozen 3, medium 2, medium 3, medium 4), pre‐amplification of tagmented DNA was carried out using the NEBNext High‐Fidelity 2× PCR Master Mix (NEB). The number of additional cycles for post‐amplification was then determined using qPCR with the NEBNext High‐Fidelity 2× PCR Master Mix (NEB) in combination with SYBR Green (Thermo Fisher Scientific). For the other spider replicates (fresh 1, frozen 1, medium 1) and the ant samples, the tagmentation reaction was purified using the M&N NucleoSpin Gel and PCR Clean‐up Kit (MACHEREY‐NAGEL) and the library amplification was tested with the Q5 High‐Fidelity 2× DNA Polymerase mix (NEB) (*Note:* It is not recommended to use Hot‐Start Polymerases as the initial extension step at 72°C will be negatively affected). In addition, for these samples (spider replicates fresh 1, frozen 1, medium 1, and ant samples), the qPCR was performed with the 5× HOT FIREPol EvaGreen qPCR Mix Plus (ROX) MIX. The final libraries were purified with either a PCR purification kit (M&N NucleoSpin Gel and PCR Clean‐up Kit, Qiagen MinElute PCR Purification Kit) (spider fresh 1, frozen 1, medium 1) or Agencourt AMPure XP beads (Beckman Coulter) using double size selection (0.5× and 1.8×) (for other spider replicates). The quantity and quality of the libraries were determined using either Agilent Bioanalyzer or Tapestation.

By comparing the different spider replicates, it is apparent that using automated counting (see above), the Q5 High‐Fidelity 2× DNA Polymerase mix for library amplification and different polymerases for library amplification and qPCR clearly affected the quality of the data negatively. Spider replicates fresh 1, frozen 1, and medium 1 showed lower quality on almost all levels: much lower peaks in the fragment size distribution, less optimal *q*‐values, more unique peaks (Supporting Information S2: Figure S2), lower mapping rates, lowest FRiP scores, the least number of peaks called (Supporting Information S6: Table S2), much less coverage in the TSS enrichment (Figure 3E). In conclusion, the first replicates performed with automated cell counting and suboptimal library amplification protocols were clearly of less quality than the other spider replicates. In addition, the spider replicates fresh 2 and fresh 3 were overamplified because the fluorescence detection point was set after the annealing step instead of after elongation during the qPCR, which led to an underestimation of the pre‐amplified library concentration. Overamplification usually generates an increased number of small fragments (see Supporting Information S2: Figure S2) and PCR duplicates (Supporting Information S6: Table 2), which can look like an over‐tagmented library (as explained in Figure 1C). Taken together, this highlights that correct nuclei numbers and integrity estimations, as well as correct library amplification, are crucial to obtain optimal ATAC‐seq data quality and might have more impact than the preservation method.

### 8.2 Bioinformatic Analysis of ATAC‐seq Data

Raw read quality controls were performed using FastQC and MultiQC (https://www.bioinformatics.babraham.ac.uk/projects/fastqc) (Ewels et al. 2016). Low‐quality reads and adapter sequences were removed using Trimmomatic (Bolger et al. 2014) for spider samples and TrimGalore (https://github.com/FelixKrueger/TrimGalore) for ant samples. Trimmed reads were blasted against the mitochondrial genome of a closely related ant species (see scripts in https://doi.org/10.25625/2TUGJU for details) for the ant sample. Less than 3.2e^−4^ of the reads were of mitochondrial origin and were therefore not removed from the dataset. Trimmed reads were aligned to the reference genomes (spider: Zhu et al. 2022, 2023, NCBI Accession: PRJNA934108) (Ant accession number: JASTWN000000000; Harrison et al. submitted) using bwa‐mem (Li and Durbin 2009) with standard settings for both species. As trimmed spider reads still contained potential mitochondrial contamination at this point of the analysis, an additional alignment step was performed against the mitochondrial genome to remove mitochondrial reads.

The resulting BAM files of both species were used for fragment size distribution analyses by Picard's *CollectInsertSizeMetrics* (https://github.com/broadinstitute/picard). Library complexity estimation was plotted using the *estimateLibComplexity* function from the ATACseqQC package in R (Ou et al. 2018). Duplicates were marked and removed using Picard's *MarkDuplicates* command. Some spider samples were found to have fragments longer than 2000 bp, which were artificially generated during mapping. These fragments were excluded for downstream analyses. To adjust the Tn5 cutting sites appropriately, the *alignmentSieve* command from deepTools (Ramírez et al. 2014) was used with the *–ATACshift* argument. Peak calling was conducted using MACS2 (Zhang et al. 2008). FRiP score calculation was performed using the *plotEnrichment* function from the deepTools package. BigWig files were generated using the *bamCoverage* command from deepTools, with BPM (bins per million) as the normalization method. Fragments shorter than 100 bp were defined as the nucleosome‐free fraction. Fragments, which mapped to gene features in the annotation file, were used to generate TSS enrichment plots. This analysis was performed using the *computeMatrix* and *plotProfile* functions from deepTools. Peak annotation was performed using the annotatePeaks.pl script from the HOMER suite (Heinz et al. 2010). Consensus peaks across samples were identified using the *multiintersect* function in bedtools (Quinlan and Hall 2010). For visual inspection of the read density and consensus peaks, we used the IGV (Thorvaldsdottir et al. 2013).

## Author Contributions

Conceptualization: Natascha Turetzek, Barbara Feldmeyer, Nico Posnien. Formal Analysis: Duğçar Ebrar Erdoğan, Barbara Feldmeyer. Funding Acquisition: Oliver Niehuis, Natascha Turetzek, Katja Nowick, Barbara Feldmeyer, Antonella Soro, Vincent Doublet, Nico Posnien. Investigation: Natascha Turetzek, Duğçar Ebrar Erdoğan, Shadi Karimifard, Liucong Ling. Methodology: Duğçar Ebrar Erdoğan, Natascha Turetzek, Nico Posnien. Project Administration: Natascha Turetzek, Nico Posnien, Barbara Feldmeyer. Resources: Natascha Turetzek, Barbara Feldmeyer, Nico Posnien, Ana Catalán. Software: Duğçar Ebrar Erdoğan, Barbara Feldmeyer, Ana Catalán. Supervision: Natascha Turetzek, Nico Posnien, Barbara Feldmeyer. Visualization: Oliver Niehuis, Nico Posnien, Natascha Turetzek. Writing – original draft preparation: Katja Nowick, Nico Posnien, Amanda Glaser‐Schmitt, Natascha Turetzek, Barbara Feldmeyer, Vincent Doublet, Antonella Soro, Ana Catalán, Duğçar Ebrar Erdoğan. Writing – review and editing: Oliver Niehuis, Antonella Soro, Natascha Turetzek, Katja Nowick, Nico Posnien, Amanda Glaser‐Schmitt, Mozhgan Khodadadi, Barbara Feldmeyer, Shadi Karimifard, Vincent Doublet, Duğçar Ebrar Erdoğan, Ana Catalán, Luisa Linke, Liucong Ling.

## Supporting information


**Supplementary Figure S1.** Images of tissue suspensions after homogenization and lysis using the protocol presented in this work (https://doi.org/10.25625/NFVW5W). **(A)** Honeybee fat body solution without any intact nuclei. The remainder of small fragments (red arrow) indicate too harsh homogenisation and lysis conditions. **(B)** Intact nuclei (green arrow) after homogenisation and lysis of spider embryos. **(C)** Stick insect solution with insufficient homogenisation and lysis conditions indicated by too much debris (black arrow). All samples were stained with Trypan Blue and imaged in an Neubauer counting chamber 0.100 mm depth.

**Supplementary Figure S2.** Post‐mapping and post‐peak calling quality assessment of ATAC‐seq data. **(A)** Fragment size distribution of replicates for spider samples using different preservation methods: fresh (green), frozen (blue), medium (orange). **(B)** Library complexity estimation for the spider replicates using different preservation methods: fresh (green), frozen (blue), medium (orange). **(C)** Comparison of lg(‐lg(*q*values)) across treatments and spider replicates. A high lg(‐lg(*q*value)) indicates that a peak is highly statistically significant (an FDR of 0.05 corresponds to 1.301 in the plots) and less likely to be a false positive. **(D)** Venn diagrams depicting the number of shared and unique peaks across spider replicates. **(E)** Venn diagrams depicting the number of shared and unique peaks between treatments of the ant samples.

**Supplementary Figure S3.** Post‐mapping and post‐peak calling quality assessment of spider ATAC‐seq data based on visual inspection using IGV. Two randomly selected genomic regions are shown in **(A)** and **(B)** respectively. Annotated genes are provided in black at the bottom of each plot. Black boxes represent exons, which are connected by straight lines representing introns. For each preservation method the read density at each genomic location is shown as grey bars and green, blue and orange boxes below indicate called consensus peaks.

**Supplementary Figure S4.** Post‐mapping and post‐peak calling quality assessment of ant ATAC‐seq data based on visual inspection using IGV. Two randomly selected genomic regions are shown in **(A)** and **(B)** respectively. Annotated genes are provided in black at the bottom of each plot. Black boxes represent exons, which are connected by straight lines representing introns. For each sample the read density at each genomic location is shown as black bars and green/blue boxes below indicate called consensus peaks.

**Supplementary Table S1.** Summary of published ATAC‐seq datasets for arthropod species excluding *Drosophila* spp. This data was used to prepare Figure 1A. The table summarises the overall research question, the tissue and preservation method used and the references.

**Supplementary Table S2.** Summary statistics for each spider ATAC‐seq replicate which were used for the calculation of the mean and standard deviation shown in Table 3.

## Data Availability

All raw reads are available at the Bioproject Accession number PRJEB87417. All scripts used to analyze and visualize the ATAC‐seq data are available in an online repository (Analysis of spider data: https://doi.org/10.25625/GKIWP6; analysis of ant data: https://doi.org/10.25625/2TUGJU). A step‐by‐step wet lab protocol is available online (https://doi.org/10.25625/NFVW5W).

## References

[jezb23305-bib-0001] Adey, A. C. 2021. “Tagmentation‐Based Single‐Cell Genomics.” Genome Research 31: 1693–1705. 10.1101/gr.275223.121. 34599003 PMC8494221

[jezb23305-bib-0002] Adey, A. , H. G. Morrison , Asan, et al. 2010. “Rapid, Low‐Input, Low‐Bias Construction of Shotgun Fragment Libraries by High‐Density In Vitro Transposition.” Genome Biology 11: R119. 10.1186/gb-2010-11-12-r119. 21143862 PMC3046479

[jezb23305-bib-0003] Altschul, S. F. , W. Gish , W. Miller , E. W. Myers , and D. J. Lipman . 1990. “Basic Local Alignment Search Tool.” Journal of Molecular Biology 215: 403–410. 10.1016/S0022-2836(05)80360-2. 2231712

[jezb23305-bib-0004] Augusto, R. C. , D. Duval , and C. Grunau . 2019. “Effects of the Environment on Developmental Plasticity and Infection Success of Schistosoma Parasites – An Epigenetic Perspective.” Frontiers in Microbiology 10: 1475. 10.3389/fmicb.2019.01475. 31354641 PMC6632547

[jezb23305-bib-0005] Augusto, R. C. , O. Rey , C. Cosseau , et al. 2021. “A Simple ATAC‐Seq Protocol for Population Epigenetics.” Wellcome Open Research 5: 121. 10.12688/wellcomeopenres.15552.2. 33521328 PMC7814285

[jezb23305-bib-0006] Basset, Y. , L. Cizek , P. Cuénoud , et al. 2012. “Arthropod Diversity in a Tropical Forest.” Science 338: 1481–1484. 10.1126/science.1226727. 23239740

[jezb23305-bib-0007] Belton, J.‐M. , R. P. McCord , J. H. Gibcus , N. Naumova , Y. Zhan , and J. Dekker . 2012. “Hi‐C: A Comprehensive Technique to Capture the Conformation of Genomes.” Methods 58: 268–276. 10.1016/j.ymeth.2012.05.001. 22652625 PMC3874846

[jezb23305-bib-0008] Bentsen, M. , P. Goymann , H. Schultheis , et al. 2020. “ATAC‐Seq Footprinting Unravels Kinetics of Transcription Factor Binding During Zygotic Genome Activation.” Nature Communications 11: 4267. 10.1038/s41467-020-18035-1. PMC7449963 32848148

[jezb23305-bib-0010] Bolger, A. M. , M. Lohse , and B. Usadel . 2014. “Trimmomatic: A Flexible Trimmer for Illumina Sequence Data.” Bioinformatics 30: 2114–2120. 10.1093/bioinformatics/btu170. 24695404 PMC4103590

[jezb23305-bib-0127] Boratyn, G. M. , J. Thierry‐Mieg , D. Thierry‐Mieg , B. Busby , and T. L. Madden . 2019. “Magic‐BLAST, an Accurate RNA‐seq Aligner for Long and Short Reads.” BMC Bioinformatics 20, no. 1: 405. 10.1186/s12859-019-2996-x. 31345161 PMC6659269

[jezb23305-bib-0011] Boyle, A. P. , S. Davis , H. P. Shulha , et al. 2008. “High‐Resolution Mapping and Characterization of Open Chromatin Across the Genome.” Cell 132: 311–322. 10.1016/j.cell.2007.12.014. 18243105 PMC2669738

[jezb23305-bib-0012] Buchberger, E. , M. Reis , T.‐H. Lu , and N. Posnien . 2019. “Cloudy With a Chance of Insights: Context Dependent Gene Regulation and Implications for Evolutionary Studies.” Genes 10, no. 7: 492. 10.3390/genes10070492. 31261769 PMC6678813

[jezb23305-bib-0013] Buenrostro, J. D. , P. G. Giresi , L. C. Zaba , H. Y. Chang , and W. J. Greenleaf . 2013. “Transposition of Native Chromatin. Native Chromatin for Fast and Sensitive Epigenomic Profiling. Sensitive Epigenomic Profiling of Open Chromatin, DNA‐Binding Proteins. Open Chromatin, DNA‐Binding Proteins and Nucleosome Position.” Nature Methods 10: 1213–1218. 10.1038/nmeth.2688. 24097267 PMC3959825

[jezb23305-bib-0014] Buenrostro, J. D. , B. Wu , H. Y. Chang , and W. J. Greenleaf . 2015. “ATAC‐Seq: A Method for Assaying Chromatin Accessibility Genome‐Wide.” Current Protocols in Molecular Biology 109: 21.29.1–21.29.9. 10.1002/0471142727.mb2129s109. PMC4374986 25559105

[jezb23305-bib-0015] Buenrostro, J. D. , B. Wu , U. M. Litzenburger , et al. 2015. “Single‐Cell Chromatin Accessibility Reveals Principles of Regulatory Variation.” Nature 523: 486–490. 10.1038/nature14590. 26083756 PMC4685948

[jezb23305-bib-0016] van der Burg, K. R. L. , J. J. Lewis , B. J. Brack , R. A. Fandino , A. Mazo‐Vargas , and R. D. Reed . 2020. “Genomic Architecture of a Genetically Assimilated Seasonal Color Pattern.” Science 370: 721–725. 10.1126/science.aaz3017. 33154142

[jezb23305-bib-0017] Burton, A. , and M.‐E. Torres‐Padilla . 2014. “Chromatin Dynamics in the Regulation of Cell Fate Allocation During Early Embryogenesis.” Nature Reviews Molecular Cell Biology 15: 723–735. 10.1038/nrm3885. 25303116

[jezb23305-bib-0018] Carroll, S. B. 2000. “Endless Forms.” Cell 101: 577–580. 10.1016/s0092-8674(00)80868-5. 10892643

[jezb23305-bib-0019] Carroll, S. B. 2008. “Evo‐Devo and an Expanding Evolutionary Synthesis: A Genetic Theory of Morphological Evolution.” Cell 134: 25–36. 10.1016/j.cell.2008.06.030. 18614008

[jezb23305-bib-0020] Catalán, A. , J. Merondun , U. Knief , and J. B. W. Wolf . 2023. “Chromatin Accessibility, Not 5mC Methylation Covaries With Partial Dosage Compensation in Crows.” PLoS Genetics 19: e1010901. https://journals.plos.org/plosgenetics/article/file?id=10.1371/journal.pgen.1010901&type=printable. 37747941 10.1371/journal.pgen.1010901 PMC10575545

[jezb23305-bib-0021] Cejas, P. , and H. W. Long . 2022. “High‐Resolution ATAC‐Seq Analysis of Frozen Clinical Tissues.” Methods in Molecular Biology 2458: 259–267. 10.1007/978-1-0716-2140-0_14. 35103972

[jezb23305-bib-0022] Corces, M. R. , J. D. Buenrostro , B. Wu , et al. 2016. “Lineage‐Specific and Single‐Cell Chromatin Accessibility Charts Human Hematopoiesis and Leukemia Evolution.” Nature Genetics 48: 1193–1203. 10.1038/ng.3646. 27526324 PMC5042844

[jezb23305-bib-0023] Corces, M. R. , A. E. Trevino , E. G. Hamilton , et al. 2017. “An Improved ATAC‐Seq Protocol Reduces Background and Enables Interrogation of Frozen Tissues.” Nature Methods 14: 959–962. 10.1038/nmeth.4396. 28846090 PMC5623106

[jezb23305-bib-0024] Corces, R. , W. J. Greenleaf , and H. Y. Chang . 2019. “Isolation of Nuclei From Frozen Tissue for ATAC‐Seq and Other Epigenomic Assays V1.” Protocols.io. 10.17504/protocols.io.6t8herw.

[jezb23305-bib-0025] Darwin Tree of Life Project Consortium . 2022. “Sequence Locally, Think Globally: The Darwin Tree of Life Project.” Proceedings of the National Academy of Sciences of the USA 119, no. 4: e2115642118. 10.1073/pnas.2115642118. 35042805 PMC8797607

[jezb23305-bib-0026] Davidson, P. L. , and A. P. Moczek . 2024. “Genome Evolution and Divergence in Cis‐Regulatory Architecture Is Associated With Condition‐Responsive Development in Horned Dung Beetles.” PLoS Genetics 20: e1011165. 10.1371/journal.pgen.1011165. 38442113 PMC10942260

[jezb23305-bib-0027] Ewels, P. , M. Magnusson , S. Lundin , and M. Käller . 2016. “MultiQC: Summarize Analysis Results for Multiple Tools and Samples in a Single Report.” Bioinformatics 32: 3047–3048. 10.1093/bioinformatics/btw354. 27312411 PMC5039924

[jezb23305-bib-0028] Fang, F. , H. Zhou , X. Feng , et al. 2022. “Gene Expression and Chromatin Conformation Differs Between Worker Bees Performing Different Tasks.” Genomics 114: 110362. 10.1016/j.ygeno.2022.110362. 35398245

[jezb23305-bib-0029] Fujiwara, S. , S. Baek , L. Varticovski , S. Kim , and G. L. Hager . 2019. “High Quality ATAC‐Seq Data Recovered From Cryopreserved Breast Cell Lines and Tissue.” Scientific Reports 9: 516. 10.1038/s41598-018-36927-7. 30679562 PMC6345852

[jezb23305-bib-0030] Gabriel, L. , T. Brůna , K. J. Hoff , et al. 2024. “BRAKER3: Fully Automated Genome Annotation Using RNA‐Seq and Protein Evidence With GeneMark‐ETP, AUGUSTUS, and TSEBRA.” Genome Research 34: 769–777. 10.1101/gr.278090.123. 38866550 PMC11216308

[jezb23305-bib-0031] García‐Alcalde, F. , K. Okonechnikov , J. Carbonell , et al. 2012. “Qualimap: Evaluating Next‐Generation Sequencing Alignment Data.” Bioinformatics 28: 2678–2679. 10.1093/bioinformatics/bts503. 22914218

[jezb23305-bib-0032] Gehrke, A. R. , and M. Srivastava . 2022. “Assessing Chromatin Accessibility During WBR in Acoels.” Methods in Molecular Biology 2450: 549–561. 10.1007/978-1-0716-2172-1_29. 35359328 PMC9761501

[jezb23305-bib-0033] Giresi, P. G. , J. Kim , R. M. McDaniell , V. R. Iyer , and J. D. Lieb . 2007. “FAIRE (Formaldehyde‐Assisted Isolation of Regulatory Elements) Isolates Active Regulatory Elements From Human Chromatin.” Genome Research 17: 877–885. 10.1101/gr.5533506. 17179217 PMC1891346

[jezb23305-bib-0034] Glastad, K. M. , B. G. Hunt , and M. A. D. Goodisman . 2019. “Epigenetics in Insects: Genome Regulation and the Generation of Phenotypic Diversity.” Annual Review of Entomology 64: 185–203. 10.1146/annurev-ento-011118-111914. 30285490

[jezb23305-bib-0035] Grandi, F. C. , H. Modi , L. Kampman , and M. R. Corces . 2022. “Chromatin Accessibility Profiling by ATAC‐Seq.” Nature Protocols 17: 1518–1552. 10.1038/s41596-022-00692-9. 35478247 PMC9189070

[jezb23305-bib-0036] Hainer, S. J. , and T. G. Fazzio . 2019. “High‐Resolution Chromatin Profiling Using CUT&RUN.” Current Protocols in Molecular Biology 126: e85. 10.1002/cpmb.85. 30688406 PMC6422702

[jezb23305-bib-0037] Hao, T. , Z. Song , M. Zhang , and L. Zhang . 2024. “Signaling Transduction Pathways and G‐Protein‐Coupled Receptors in Different Stages of the Embryonic Diapause Termination Process in Artemia.” Current Issues in Molecular Biology 46: 3676–3693. 10.3390/cimb46040229. 38666959 PMC11049050

[jezb23305-bib-0038] Heinz, S. , C. Benner , N. Spann , et al. 2010. “Simple Combinations of Lineage‐Determining Transcription Factors Prime Cis‐Regulatory Elements Required for Macrophage and B Cell Identities.” Molecular Cell 38: 576–589. 10.1016/j.molcel.2010.05.004. 20513432 PMC2898526

[jezb23305-bib-0039] Hentges, L. D. , M. J. Sergeant , C. B. Cole , D. J. Downes , J. R. Hughes , and S. Taylor . 2022. “LanceOtron: A Deep Learning Peak Caller for Genome Sequencing Experiments.” Bioinformatics 38: 4255–4263. 10.1093/bioinformatics/btac525. 35866989 PMC9477537

[jezb23305-bib-0040] Hill, M. S. , P. Vande Zande , and P. J. Wittkopp . 2021. “Molecular and Evolutionary Processes Generating Variation in Gene Expression.” Nature Reviews Genetics 22: 203–215. 10.1038/s41576-020-00304-w. PMC7981258 33268840

[jezb23305-bib-0041] Ibrahim, M. M. , S. A. Lacadie , and U. Ohler . 2015. “JAMM: A Peak Finder for Joint Analysis of NGS Replicates.” Bioinformatics 31: 48–55. 10.1093/bioinformatics/btu568. 25223640

[jezb23305-bib-0042] Iwasaki‐Yokozawa, S. , R. Nanjo , Y. Akiyama‐Oda , and H. Oda . 2022. “Lineage‐Specific, Fast‐Evolving GATA‐Like Gene Regulates Zygotic Gene Activation to Promote Endoderm Specification and Pattern Formation in the Theridiidae Spider.” BMC Biology 20: 223. 10.1186/s12915-022-01421-0. 36203191 PMC9535882

[jezb23305-bib-0043] Jones, B. M. , V. D. Rao , T. Gernat , et al. 2020. “Individual Differences in Honey Bee Behavior Enabled by Plasticity in Brain Gene Regulatory Networks.” eLife 9: e62850. 10.7554/eLife.62850. 33350385 PMC7755388

[jezb23305-bib-0044] Kaya‐Okur, H. S. , S. J. Wu , C. A. Codomo , et al. 2019. “CUT&Tag for Efficient Epigenomic Profiling of Small Samples and Single Cells.” Nature Communications 10: 1930. 10.1038/s41467-019-09982-5. PMC6488672 31036827

[jezb23305-bib-0045] Kharchenko, P. V. , M. Y. Tolstorukov , and P. J. Park . 2008. “Design and Analysis of ChIP‐Seq Experiments for DNA‐Binding Proteins.” Nature Biotechnology 26: 1351–1359. 10.1038/nbt.1508. PMC2597701 19029915

[jezb23305-bib-0046] Khodursky, S. , E. B. Zheng , N. Svetec , et al. 2023. “The Evolution and Mutational Robustness of Chromatin Accessibility in *Drosophila* .” Genome Biology 24: 232. 10.1186/s13059-023-03079-5. 37845780 PMC10578003

[jezb23305-bib-0047] King, M. C. , and A. C. Wilson . 1975. “Evolution at Two Levels in Humans and Chimpanzees.” Science 188: 107–116. 10.1126/science.1090005. 1090005

[jezb23305-bib-0048] Kissane, S. , V. Dhandapani , and L. Orsini . 2021. “Protocol for Assay of Transposase Accessible Chromatin Sequencing in Non‐Model Species.” STAR Protocols 2: 100341. 10.1016/j.xpro.2021.100341. 33659905 PMC7896190

[jezb23305-bib-0049] Klemm, S. L. , Z. Shipony , and W. J. Greenleaf . 2019. “Chromatin Accessibility and the Regulatory Epigenome.” Nature Reviews Genetics 20: 207–220. 10.1038/s41576-018-0089-8. 30675018

[jezb23305-bib-0050] Kong, X. , G. Wei , N. Chen , et al. 2020. “Dynamic Chromatin Accessibility Profiling Reveals Changes in Host Genome Organization in Response to Baculovirus Infection.” PLoS Pathogens 16: e1008633. 10.1371/journal.ppat.1008633. 32511266 PMC7326278

[jezb23305-bib-0051] Langmead, B. , and S. L. Salzberg . 2012. “Fast Gapped‐Read Alignment With Bowtie 2.” Nature Methods 9: 357–359. 10.1038/nmeth.1923. 22388286 PMC3322381

[jezb23305-bib-0052] Lewis, J. J. , R. C. Geltman , P. C. Pollak , et al. 2019. “Parallel Evolution of Ancient, Pleiotropic Enhancers Underlies Butterfly Wing Pattern Mimicry.” Proceedings of the National Academy of Sciences 116: 24174–24183. 10.1073/pnas.1907068116. PMC6883815 31712408

[jezb23305-bib-0053] Li, A. , Z. Song , M. Zhang , et al. 2024. “Integrating ATAC‐Seq and RNA‐Seq Reveals the Signal Regulation Involved in the Artemia Embryonic Reactivation Process.” Genes 15: 1083. 10.3390/genes15081083. 39202442 PMC11353689

[jezb23305-bib-0054] Li, H. 2013. “Aligning Sequence Reads, Clone Sequences and Assembly Contigs With BWA‐MEM.” arXiv [q‐bioGN]. http://arxiv.org/abs/1303.3997.

[jezb23305-bib-0055] Li, H. , and R. Durbin . 2009. “Fast and Accurate Short Read Alignment With Burrows‐Wheeler Transform.” Bioinformatics 25: 1754–1760. 10.1093/bioinformatics/btp324. 19451168 PMC2705234

[jezb23305-bib-0056] Li, H. , B. Handsaker , A. Wysoker , et al. 2009. “The Sequence Alignment/Map Format and SAMtools.” Bioinformatics 25: 2078–2079. 10.1093/bioinformatics/btp352. 19505943 PMC2723002

[jezb23305-bib-0057] Li, N. , K. Jin , Y. Bai , H. Fu , L. Liu , and B. Liu . 2020. “Tn5 Transposase Applied in Genomics Research.” International Journal of Molecular Sciences 21: 8329. 10.3390/ijms21218329. 33172005 PMC7664229

[jezb23305-bib-0058] Li, Z. , M. H. Schulz , T. Look , M. Begemann , M. Zenke , and I. G. Costa . 2019. “Identification of Transcription Factor Binding Sites Using ATAC‐Seq.” Genome Biology 20: 45. 10.1186/s13059-019-1642-2. 30808370 PMC6391789

[jezb23305-bib-0059] Liu, C. , M. Wang , X. Wei , et al. 2019. “An ATAC‐Seq Atlas of Chromatin Accessibility in Mouse Tissues.” Scientific Data 6: 65. 10.1038/s41597-019-0071-0. 31110271 PMC6527694

[jezb23305-bib-0060] Lowe, R. , M. Wojciechowski , N. Ellis , and P. J. Hurd . 2022. “Chromatin Accessibility‐Based Characterisation of Brain Gene Regulatory Networks in Three Distinct Honey Bee Polyphenisms.” Nucleic Acids Research 50: 11550–11562. 10.1093/nar/gkac992. 36330958 PMC9723623

[jezb23305-bib-0061] Ma, S. , and Y. Zhang . 2020. “Profiling Chromatin Regulatory Landscape: Insights Into the Development of ChIP‐seq and ATAC‐Seq.” Molecular Biomedicine 1: 9. 10.1186/s43556-020-00009-w. 34765994 PMC7546943

[jezb23305-bib-0062] Marx, M. T. , P. Guhmann , and P. Decker . 2012. “Adaptations and Predispositions of Different Middle European Arthropod Taxa (Collembola, Araneae, Chilopoda, Diplopoda) to Flooding and Drought Conditions.” Animals 2: 564–590. 10.3390/ani2040564. 26487164 PMC4494283

[jezb23305-bib-0063] Matys, V. 2006. “TRANSFAC(R) and Its Module TRANSCompel(R): Transcriptional Gene Regulation in Eukaryotes.” Nucleic Acids Research 34: D108–D110. 10.1093/nar/gkj143. 16381825 PMC1347505

[jezb23305-bib-0064] Mau, C. , H. Rudolf , F. Strobl , et al. 2023. “How Enhancers Regulate Wavelike Gene Expression Patterns.” eLife 12: e84969. 10.7554/eLife.84969. 37432987 PMC10368423

[jezb23305-bib-0065] Mazo‐Vargas, A. , A. M. Langmüller , A. Wilder , et al. 2022. “Deep Cis‐Regulatory Homology of the Butterfly Wing Pattern Ground Plan.” Science 378: 304–308. 10.1126/science.abi9407. 36264807

[jezb23305-bib-0066] Mc Cartney, A. M. , G. Formenti , and A. Mouton , et al. 2024. “The European Reference Genome Atlas: Piloting a Decentralised Approach to Equitable Biodiversity Genomics.” NPJ Biodiversity 3: 28. 10.1038/s44185-024-00054-6. 39289538 PMC11408602

[jezb23305-bib-0067] Mittmann, B. , and C. Wolff . 2012. “Embryonic Development and Staging of the Cobweb Spider *Parasteatoda tepidariorum* C. L. Koch, 1841 (syn.: *Achaearanea tepidariorum*; Araneomorphae; Theridiidae).” Development Genes and Evolution 222: 189–216. 10.1007/s00427-012-0401-0. 22569930

[jezb23305-bib-0068] Murugesan, S. N. , H. Connahs , Y. Matsuoka , et al. 2022. “Butterfly Eyespots Evolved via Cooption of an Ancestral Gene‐Regulatory Network That Also Patterns Antennae, Legs, and Wings.” Proceedings of the National Academy of Sciences of the United States of America 119: e2108661119. 10.1073/pnas.2108661119. 35169073 PMC8872758

[jezb23305-bib-0069] Murugesan, S. N. , and A. Monteiro . 2023. “Butterfly Eyespots Exhibit Unique Patterns of Open Chromatin.” F1000Research 12: 1428. 10.12688/f1000research.133789.1. 38778811 PMC11109672

[jezb23305-bib-0070] Nair, V. D. , M. Vasoya , V. Nair , et al. 2022. “Optimization of the Omni‐ATAC Protocol to Chromatin Accessibility Profiling in Snap‐Frozen Rat Adipose and Muscle Tissues.” MethodsX 9: 101681. 10.1016/j.mex.2022.101681. 35464805 PMC9027329

[jezb23305-bib-0071] Natsidis, P. , P. Kapli , P. H. Schiffer , and M. J. Telford . 2021. “Systematic Errors in Orthology Inference and Their Effects on Evolutionary Analyses.” iScience 24: 102110. 10.1016/j.isci.2021.102110. 33659875 PMC7892920

[jezb23305-bib-0072] Ou, J. , H. Liu , J. Yu , et al. 2018. “ATACseqQC: A Bioconductor Package for Post‐Alignment Quality Assessment of ATAC‐Seq Data.” BMC Genomics 19: 169. 10.1186/s12864-018-4559-3. 29490630 PMC5831847

[jezb23305-bib-0073] Pallarès‐Albanell, J. , L. Ortega‐Flores , T. Senar‐Serra , et al. 2024. “Gene Regulatory Dynamics During the Development of a Paleopteran Insect, the Mayfly *Cloeon dipterum* .” Development (Cambridge, England) 151, no. 20: dev203017. 10.1242/dev.203017. 39324209 PMC11491810

[jezb23305-bib-0074] Peng, S. , R. Bellone , J. L. Petersen , T. S. Kalbfleisch , and C. J. Finno . 2021. “Successful ATAC‐Seq From Snap‐Frozen Equine Tissues.” Frontiers in Genetics 12: 641788. 10.3389/fgene.2021.641788. 34220931 PMC8242358

[jezb23305-bib-0075] Piovesan, A. , F. Antonaros , L. Vitale , P. Strippoli , M. C. Pelleri , and M. Caracausi . 2019. “Human Protein‐Coding Genes and Gene Feature Statistics in 2019.” BMC Research Notes 12: 315. 10.1186/s13104-019-4343-8. 31164174 PMC6549324

[jezb23305-bib-0076] Pranzatelli, T. J. F. , D. G. Michael , and J. A. Chiorini . 2018. “ATAC2GRN: Optimized ATAC‐Seq and DNase1‐Seq Pipelines for Rapid and Accurate Genome Regulatory Network Inference.” BMC Genomics 19: 563. 10.1186/s12864-018-4943-z. 30064353 PMC6069842

[jezb23305-bib-0077] Prescott, S. L. , R. Srinivasan , M. C. Marchetto , et al. 2015. “Enhancer Divergence and Cis‐Regulatory Evolution in the Human and Chimp Neural Crest.” Cell 163: 68–83. 10.1016/j.cell.2015.08.036. 26365491 PMC4848043

[jezb23305-bib-0078] Quinlan, A. R. , and I. M. Hall . 2010. “BEDTools: A Flexible Suite of Utilities for Comparing Genomic Features.” Bioinformatics 26: 841–842. 10.1093/bioinformatics/btq033. 20110278 PMC2832824

[jezb23305-bib-0079] Ramírez, F. , F. Dündar , S. Diehl , B. A. Grüning , and T. Manke . 2014. “deepTools: A Flexible Platform for Exploring Deep‐Sequencing Data.” Nucleic Acids Research 42: W187–W191. 10.1093/nar/gku365. 24799436 PMC4086134

[jezb23305-bib-0080] Ramírez, F. , D. P. Ryan , B. Grüning , et al. 2016. “deepTools2: A Next Generation Web Server for Deep‐Sequencing Data Analysis.” Nucleic Acids Research 44: W160–W165. 10.1093/nar/gkw257. 27079975 PMC4987876

[jezb23305-bib-0081] Rauluseviciute, I. , R. Riudavets‐Puig , R. Blanc‐Mathieu , et al. 2024. “JASPAR 2024: 20th Anniversary of the Open‐Access Database of Transcription Factor Binding Profiles.” Nucleic Acids Research 52: D174–D182. 10.1093/nar/gkad1059. 37962376 PMC10767809

[jezb23305-bib-0082] Rhoads, A. , and K. F. Au . 2015. “PacBio Sequencing and Its Applications.” Genomics, Proteomics & Bioinformatics 13: 278–289. 10.1016/j.gpb.2015.08.002. PMC4678779 26542840

[jezb23305-bib-0083] Rochette, N. C. , A. G. Rivera‐Colón , J. Walsh , T. J. Sanger , S. C. Campbell‐Staton , and J. M. Catchen . 2023. “On the Causes, Consequences, and Avoidance of PCR Duplicates: Towards a Theory of Library Complexity.” Molecular Ecology Resources 23: 1299–1318. 10.1111/1755-0998.13800. 37062860

[jezb23305-bib-0084] Ruggieri, A. A. , L. Livraghi , J. J. Lewis , et al. 2022. “A Butterfly Pan‐Genome Reveals That a Large Amount of Structural Variation Underlies the Evolution of Chromatin Accessibility.” Genome Research 32: 1862–1875. 10.1101/gr.276839.122. 36109150 PMC9712634

[jezb23305-bib-0085] Sana, J. , P. Faltejskova , M. Svoboda , and O. Slaby . 2012. “Novel Classes of Non‐Coding RNAs and Cancer.” Journal of Translational Medicine 10: 103. 10.1186/1479-5876-10-103. 22613733 PMC3434024

[jezb23305-bib-0086] Satpathy, A. T. , N. Saligrama , J. D. Buenrostro , et al. 2018. “Transcript‐Indexed ATAC‐Seq for Precision Immune Profiling.” Nature Medicine 24: 580–590. 10.1038/s41591-018-0008-8. PMC5948148 29686426

[jezb23305-bib-0087] Schmitz, R. J. , A. P. Marand , X. Zhang , et al. 2022. “Quality Control and Evaluation of Plant Epigenomics Data.” Plant Cell 34: 503–513. 10.1093/plcell/koab255. 34648025 PMC8773985

[jezb23305-bib-0088] Schulze, S. R. , and L. L. Wallrath . 2007. “Gene Regulation by Chromatin Structure: Paradigms Established in *Drosophila melanogaster* .” Annual Review of Entomology 52: 171–192. 10.1146/annurev.ento.51.110104.151007. 16881818

[jezb23305-bib-0089] Segorbe, D. , D. Wilkinson , A. Mizeranschi , et al. 2018. “An Optimized FAIRE Procedure for Low Cell Numbers in Yeast.” Yeast 35: 507–512. 10.1002/yea.3316. 29577419 PMC6099244

[jezb23305-bib-0090] Senji Laxme, R. R. , V. Suranse , and K. Sunagar . 2019. “Arthropod Venoms: Biochemistry, Ecology and Evolution.” Toxicon 158: 84–103. 10.1016/j.toxicon.2018.11.433. 30529476

[jezb23305-bib-0091] Shen, X. , X. Wang , N. Yang , et al. 2023. “Characteristics of the Accessible Chromatin Landscape and Transcriptome Under Different Temperature Stresses in *Bemisia tabaci* .” Genes 14: 1978. 10.3390/genes14101978. 37895327 PMC10606294

[jezb23305-bib-0092] Sun, D. A. , J. V. Bredeson , H. S. Bruce , and N. H. Patel . 2022. “Identification and Classification of Cis‐Regulatory Elements in the Amphipod Crustacean *Parhyale hawaiensis* .” Development 149, no. 11: dev200793. 10.1242/dev.200793. 35608283

[jezb23305-bib-0093] Tarbell, E. D. , and T. Liu . 2019. “HMMRATAC: A Hidden Markov ModeleR for ATAC‐Seq.” Nucleic Acids Research 47: e91. 10.1093/nar/gkz533. 31199868 PMC6895260

[jezb23305-bib-0094] Tendolkar, A. , A. Mazo‐Vargas , L. Livraghi , et al. 2024. “Cis‐Regulatory Modes of Ultrabithorax Inactivation in Butterfly Forewings.” eLife 12: RP90846. 10.7554/elife.90846.3. 38261357 PMC10945631

[jezb23305-bib-0095] Thorvaldsdottir, H. , J. T. Robinson , and J. P. Mesirov . 2013. “Integrative Genomics Viewer (IGV): High‐Performance Genomics Data Visualization and Exploration.” Briefings in Bioinformatics 14: 178–192. 10.1093/bib/bbs017. 22517427 PMC3603213

[jezb23305-bib-0096] Vaisvila, R. , V. K. C. Ponnaluri , Z. Sun , et al. 2021. “Enzymatic Methyl Sequencing Detects DNA Methylation at Single‐Base Resolution From Picograms of DNA.” Genome Research 31: 1280–1289. 10.1101/gr.266551.120. 34140313 PMC8256858

[jezb23305-bib-0097] Van Belleghem, S. M. , A. A. Ruggieri , and C. Concha , et al. 2023. “High Level of Novelty Under the Hood of Convergent Evolution.” Science 379: 1043–1049. 10.1126/science.ade0004. 36893249 PMC11000492

[jezb23305-bib-0098] Van den Berge, K. , H.‐J. Chou , and H. Roux de Bézieux , et al. 2022. “Normalization Benchmark of ATAC‐Seq Datasets Shows the Importance of Accounting for GC‐Content Effects.” Cell Reports Methods 2: 100321. 10.1016/j.crmeth.2022.100321. 36452861 PMC9701614

[jezb23305-bib-0099] Villani, M. G. , L. L. Allee , A. Díaz , and P. S. Robbins . 1999. “Adaptive Strategies of Edaphic Arthropods.” Annual Review of Entomology 44: 233–256. 10.1146/annurev.ento.44.1.233. 15012373

[jezb23305-bib-0100] Vorontsov, I. E. , I. A. Eliseeva , A. Zinkevich , et al. 2024. “HOCOMOCO in 2024: A Rebuild of the Curated Collection of Binding Models for Human and Mouse Transcription Factors.” Nucleic Acids Research 52: D154–D163. 10.1093/nar/gkad1077. 37971293 PMC10767914

[jezb23305-bib-0101] Vu, H. T. H. , Y. Zhang , G. Tuteja , and K. S. Dorman . 2023. “Unsupervised Contrastive Peak Caller for ATAC‐Seq.” Genome Research 33: 1133–1144. 10.1101/gr.277677.123. 37217250 PMC10538491

[jezb23305-bib-0102] Wan, W.‐T. , Z.‐W. Dong , Y.‐D. Ren , et al. 2021. “Chromatin Accessibility Profiling Provides Insights Into Larval Cuticle Color and Adult Longevity in Butterflies.” Zoological Research 42: 614–619. 10.24272/j.issn.2095-8137.2021.117. 34402607 PMC8455473

[jezb23305-bib-0103] Wan, W.‐T. , P. Hu , Z. Chang , et al. 2022. “Genome‐Wide Survey of Open Chromatin Regions in Two Swallowtail Butterflies *Papilio machaon* and *P. bianor* .” Archives of Insect Biochemistry and Physiology 111: e21952. 10.1002/arch.21952. 35909310

[jezb23305-bib-0104] Wang, G. , G. Jiang , R. Peng , et al. 2024. “Multi‐Omics Integrative Analysis Revealed Characteristic Changes in Blood Cell Immunity and Amino Acid Metabolism in a Silkworm Model of Hyperproteinemia.” International Journal of Biological Macromolecules 258: 128809. 10.1016/j.ijbiomac.2023.128809. 38128801

[jezb23305-bib-0105] Wang, M. , Y. Liu , T. Wen , et al. 2020. “Chromatin Accessibility and Transcriptome Landscapes of *Monomorium pharaonis* Brain.” Scientific Data 7: 217. 10.1038/s41597-020-0556-x. 32641764 PMC7343836

[jezb23305-bib-0106] Wang, Y. , Y. Zhao , A. Bollas , Y. Wang , and K. F. Au . 2021. “Nanopore Sequencing Technology, Bioinformatics and Applications.” Nature Biotechnology 39: 1348–1365. 10.1038/s41587-021-01108-x. PMC8988251 34750572

[jezb23305-bib-0107] Wang, Z. , M. Gerstein , and M. Snyder . 2009. “RNA‐Seq: A Revolutionary Tool for Transcriptomics.” Nature Reviews Genetics 10: 57–63. 10.1038/nrg2484. PMC2949280 19015660

[jezb23305-bib-0108] Wittkopp, P. J. , and G. Kalay . 2011. “Cis‐Regulatory Elements: Molecular Mechanisms and Evolutionary Processes Underlying Divergence.” Nature Reviews Genetics 13: 59–69. 10.1038/nrg3095. 22143240

[jezb23305-bib-0109] Wray, G. A. 2003. “The Evolution of Transcriptional Regulation in Eukaryotes.” Molecular Biology and Evolution 20: 1377–1419. 10.1093/molbev/msg140. 12777501

[jezb23305-bib-0110] Wray, G. A. 2007. “The Evolutionary Significance of Cis‐Regulatory Mutations.” Nature Reviews Genetics 8: 206–216. 10.1038/nrg2063. 17304246

[jezb23305-bib-0111] Wu, X. , M. Lu , D. Yun , et al. 2022. “Single‐Cell ATAC‐Seq Reveals Cell Type‐Specific Transcriptional Regulation and Unique Chromatin Accessibility in Human Spermatogenesis.” Human Molecular Genetics 31: 321–333. 10.1093/hmg/ddab006. 33438010

[jezb23305-bib-0112] Xin, Y. , Y. Le Poul , L. Ling , et al. 2020. “Ancestral and Derived Transcriptional Enhancers Share Regulatory Sequence and a Pleiotropic Site Affecting Chromatin Accessibility.” Proceedings of the National Academy of Sciences 117: 20636–20644. 10.1073/pnas.2004003117. PMC7456186 32778581

[jezb23305-bib-0113] Xu, B. , X. Li , X. Gao , et al. 2022. “DeNOPA: Decoding Nucleosome Positions Sensitively With Sparse ATAC‐Seq Data.” Briefings in Bioinformatics 23, no. 1: bbab469. 10.1093/bib/bbab469. 34875002

[jezb23305-bib-0114] Yan, F. , D. R. Powell , D. J. Curtis , and N. C. Wong . 2020. “From Reads to Insight: A Hitchhiker's Guide to ATAC‐Seq Data Analysis.” Genome Biology 21: 22. 10.1186/s13059-020-1929-3. 32014034 PMC6996192

[jezb23305-bib-0115] Zhang, G. , Y. Fu , L. Yang , et al. 2024. “Construction of Single‐Cell Cross‐Species Chromatin Accessibility Landscapes With Combinatorial‐Hybridization‐Based ATAC‐Seq.” Developmental Cell 59: 793–811.e8. 10.1016/j.devcel.2024.01.015. 38330939

[jezb23305-bib-0116] Zhang, H. , T. Lu , S. Liu , et al. 2021. “Comprehensive Understanding of Tn5 Insertion Preference Improves Transcription Regulatory Element Identification.” NAR Genomics & Bioinformatics 3: lqab094. 10.1093/nargab/lqab094. 34729473 PMC8557372

[jezb23305-bib-0117] Zhang, H. , M. E. Rice , J. W. Alvin , et al. 2022. “Extensive Evaluation of ATAC‐Seq Protocols for Native or Formaldehyde‐Fixed Nuclei.” BMC Genomics 23: 214. 10.1186/s12864-021-08266-x. 35296236 PMC8928671

[jezb23305-bib-0118] Zhang, Y. , X. J. He , A. B. Barron , et al. 2023. “The Diverging Epigenomic Landscapes of Honeybee Queens and Workers Revealed by Multiomic Sequencing.” Insect Biochemistry and Molecular Biology 155: 103929. 10.1016/j.ibmb.2023.103929. 36906046

[jezb23305-bib-0119] Zhang, Y. , T. Liu , C. A. Meyer , et al. 2008. “Model‐Based Analysis of ChIP‐Seq (MACS).” Genome Biology 9: R137. 10.1186/gb-2008-9-9-r137. 18798982 PMC2592715

[jezb23305-bib-0120] Zhang, Z.‐Q. 2011. “Animal Biodiversity: An Introduction to Higher‐Level Classification and Taxonomic Richness.” Zootaxa 3148: 7. 10.11646/zootaxa.3148.1.3. 26146682

[jezb23305-bib-0121] Zhao, S. , Y. Li , G. Chen , X. Wang , N. Chen , and X. Wu . 2023. “Genome‐Wide Chromatin Interaction Profiling Reveals a Vital Role of Super‐Enhancers and Rearrangements in Host Enhancer Contacts During BmNPV Infection.” Genome Research 33: 1958–1974. 10.1101/gr.277931.123. 37871969 PMC10760458

[jezb23305-bib-0122] Zhao, Y. , X. Zhang , Z. Song , et al. 2020. “Bibliometric Analysis of ATAC‐Seq and Its Use in Cancer Biology via Nucleic Acid Detection.” Frontiers in Medicine 7: 584728. 10.3389/fmed.2020.584728. 33224964 PMC7670091

[jezb23305-bib-0123] Zhou, B. , P. Hu , G. Liu , et al. 2024. “Evolutionary Patterns and Functional Effects of 3D Chromatin Structures in Butterflies With Extensive Genome Rearrangements.” Nature Communications 15: 6303. 10.1038/s41467-024-50529-0. PMC11282110 39060230

[jezb23305-bib-0128] Zhu, B. , P. Jin , Y. Zhang , et al. 2022. “Data and Code for the Comparison of Gene Expression Patterns Between the Spider Venom Glands and Other Tissues[DS/OL]. V4.” Science Data Bank. https://cstr.cn/31253.11.sciencedb.o00019.00014.

[jezb23305-bib-0124] Zhu, B. , P. Jin , Y. Zhang , Y. Shen , W. Wang , and S. Li . 2023. “Genomic and Transcriptomic Analyses Support a Silk Gland Origin of Spider Venom Glands.” BMC Biology 21: 82. 10.1186/s12915-023-01581-7. 37055766 PMC10099834

[jezb23305-bib-0125] Zu, S. , Y. E. Li , K. Wang , et al. 2023. “Single‐Cell Analysis of Chromatin Accessibility in the Adult Mouse Brain.” Nature 624: 378–389. 10.1038/s41586-023-06824-9. 38092917 PMC10719105

