# Supplementary figures and images for: ATAC‐seq in Emerging Model Organisms: Challenges and Strategies
Source: J Exp Zool B Mol Dev Evol. 2025 Jun 1;344(7):394–414. doi: 10.1002/jez.b.23305 (PMC12576391; doi:10.1002/jez.b.23305)

**A**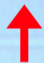

50  $\mu\text{m}$

**B**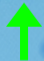

250  $\mu\text{m}$

**C**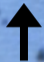

Supplement: Supplementary file 1 — Supplementary Figure S1. Images of tissue suspensions after homogenization and lysis using the protocol presented in this work (https://doi.org/10.25625/NFVW5W). (A) Honeybee fat body solution without any intact nuclei. The remainder of small fragments (red arrow) indicate too harsh homogenisation and lysis conditions. (B) Intact nuclei (green arrow) after homogenisation and lysis of spider embryos. (C) Stick insect solution with insufficient homogenisation and lysis conditions indicated by too much debris (black arrow). All samples were stained with Trypan Blue and imaged in an Neubauer counting chamber 0.100 mm depth. [file JEZ-344-394-s006.pdf]

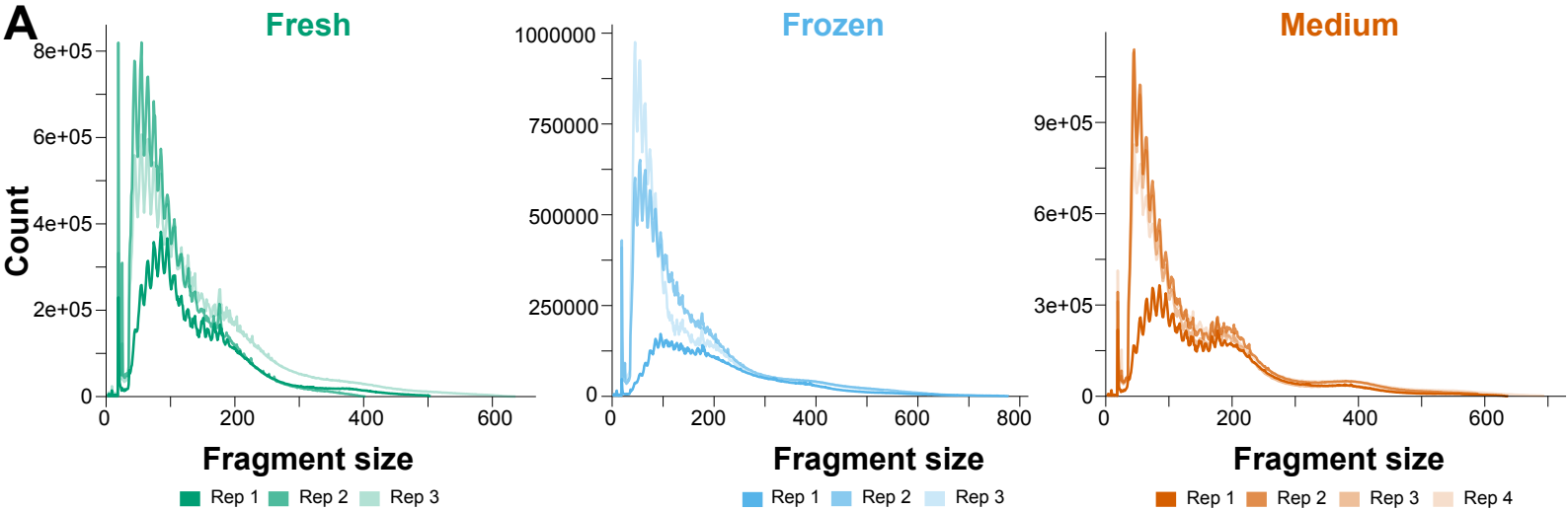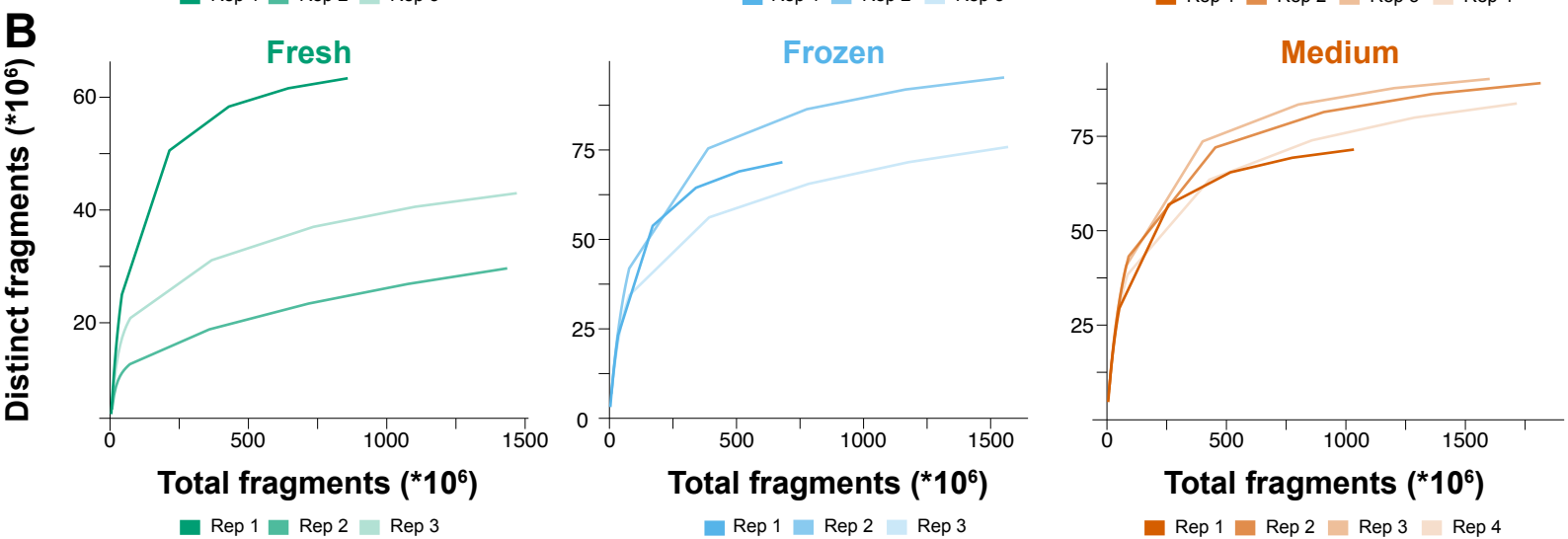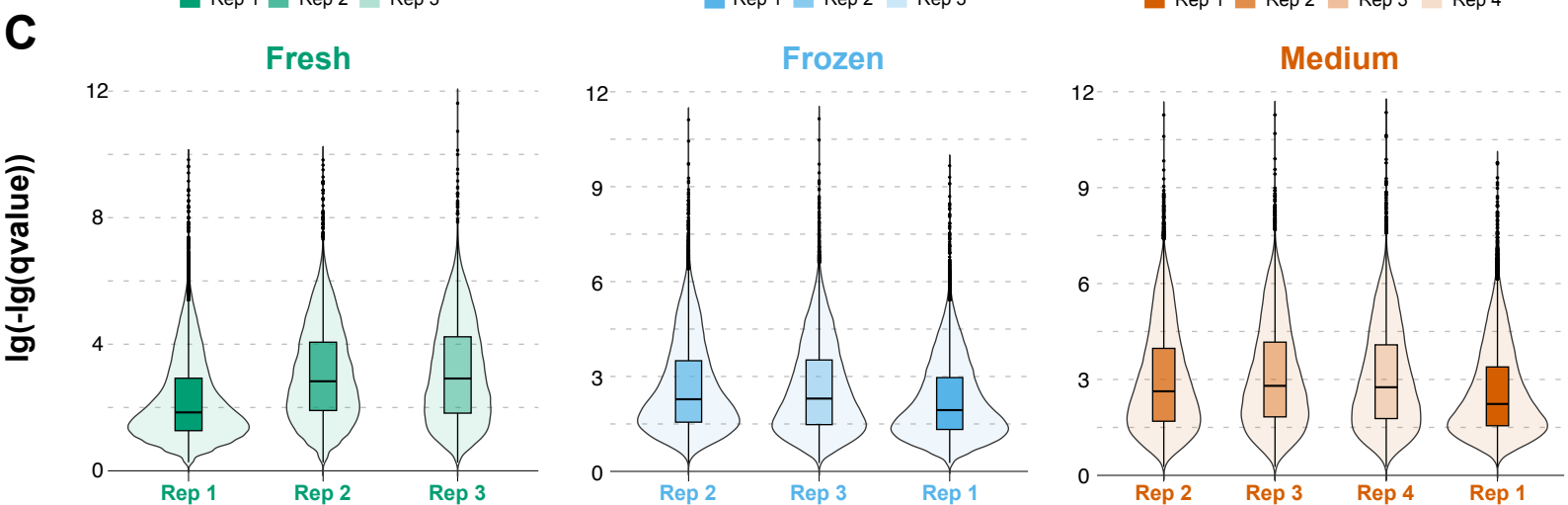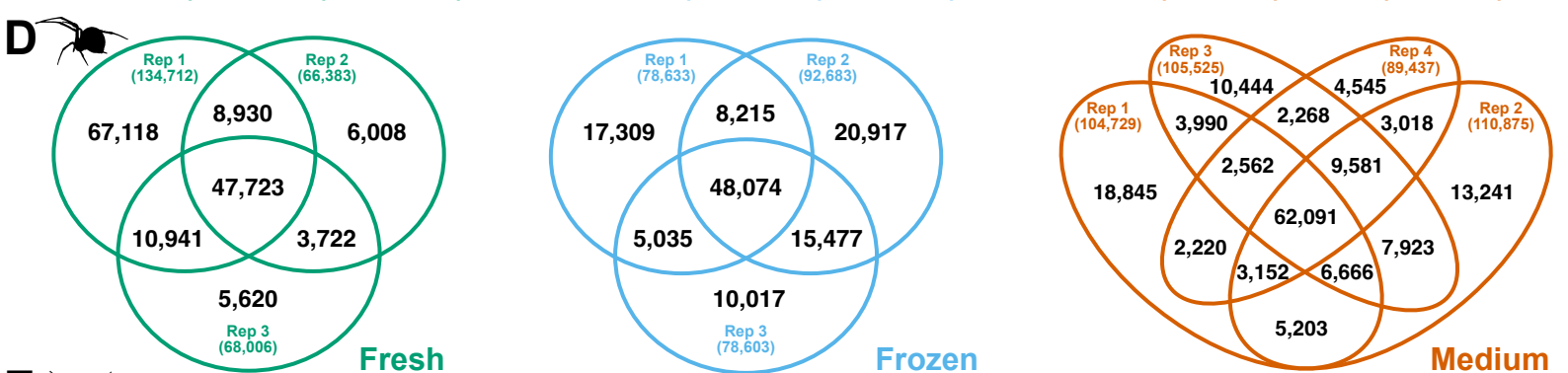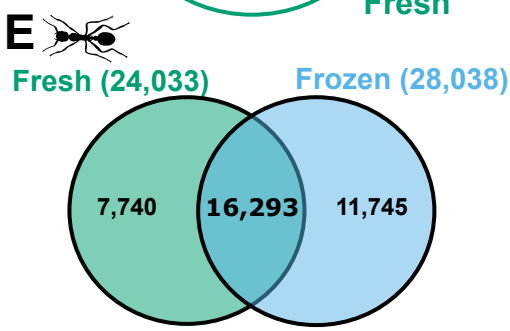

Supplement: Supplementary file 2 — Supplementary Figure S2. Post‐mapping and post‐peak calling quality assessment of ATAC‐seq data. (A) Fragment size distribution of replicates for spider samples using different preservation methods: fresh (green), frozen (blue), medium (orange). (B) Library complexity estimation for the spider replicates using different preservation methods: fresh (green), frozen (blue), medium (orange). (C) Comparison of lg(‐lg(qvalues)) across treatments and spider replicates. A high lg(‐lg(qvalue)) indicates that a peak is highly statistically significant (an FDR of 0.05 corresponds to 1.301 in the plots) and less likely to be a false positive. (D) Venn diagrams depicting the number of shared and unique peaks across spider replicates. (E) Venn diagrams depicting the number of shared and unique peaks between treatments of the ant samples. [file JEZ-344-394-s002.pdf]

**A**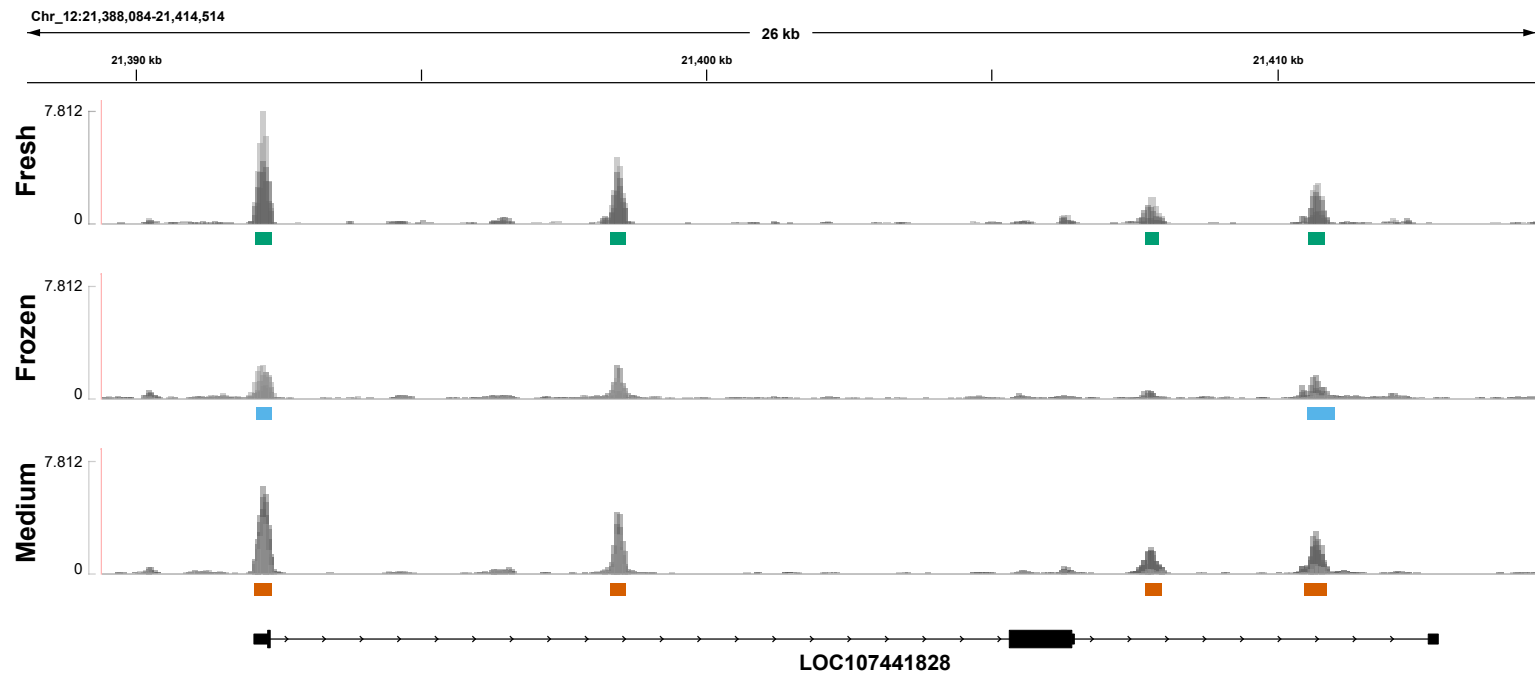**B**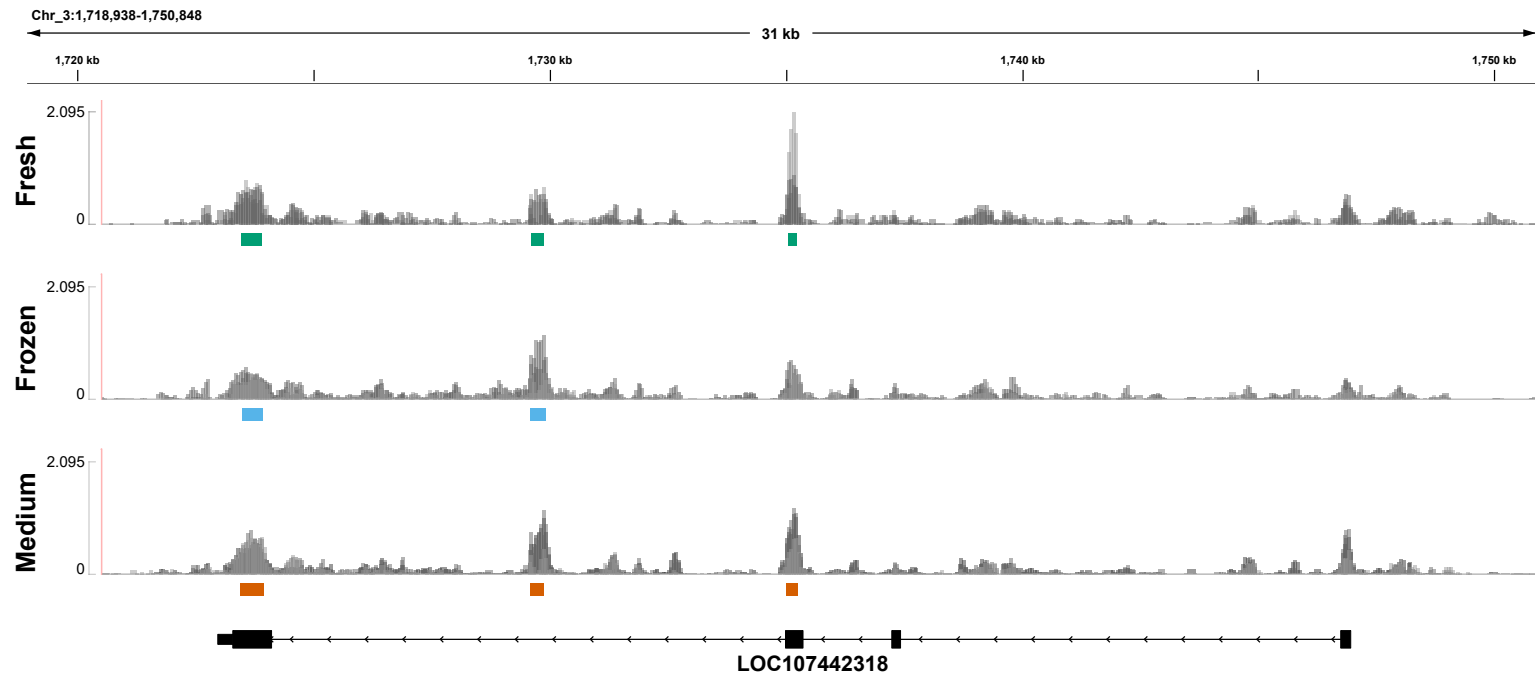

Supplement: Supplementary file 3 — Supplementary Figure S3. Post‐mapping and post‐peak calling quality assessment of spider ATAC‐seq data based on visual inspection using IGV. Two randomly selected genomic regions are shown in (A) and (B) respectively. Annotated genes are provided in black at the bottom of each plot. Black boxes represent exons, which are connected by straight lines representing introns. For each preservation method the read density at each genomic location is shown as grey bars and green, blue and orange boxes below indicate called consensus peaks. [file JEZ-344-394-s004.pdf]

**A**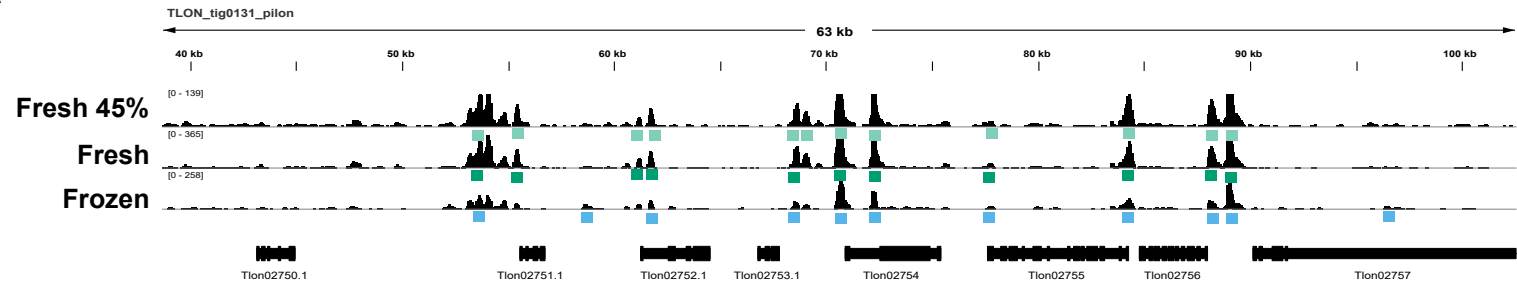**B**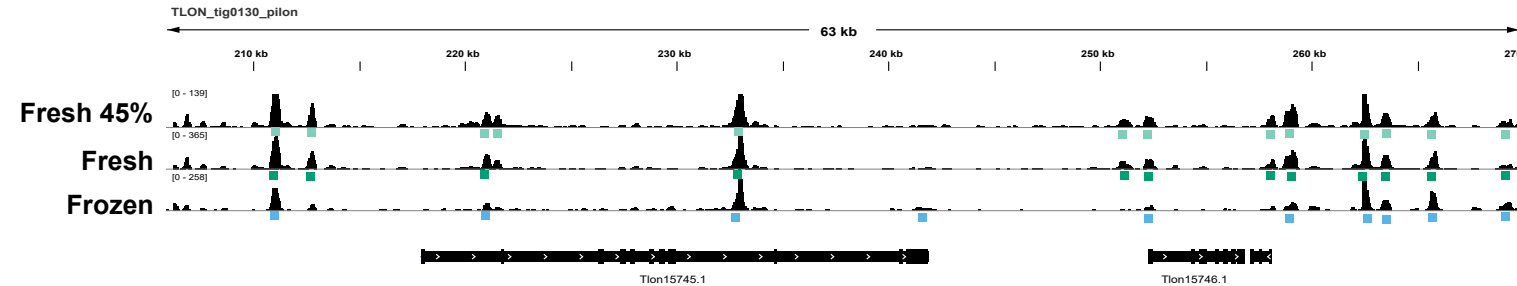

Supplement: Supplementary file 4 — Supplementary Figure S4. Post‐mapping and post‐peak calling quality assessment of ant ATAC‐seq data based on visual inspection using IGV. Two randomly selected genomic regions are shown in (A) and (B) respectively. Annotated genes are provided in black at the bottom of each plot. Black boxes represent exons, which are connected by straight lines representing introns. For each sample the read density at each genomic location is shown as black bars and green/blue boxes below indicate called consensus peaks. [file JEZ-344-394-s003.pdf]
